# Enzyme Kinetics by Isothermal Titration Calorimetry: Allostery, Inhibition, and Dynamics

**DOI:** 10.3389/fmolb.2020.583826

**Published:** 2020-10-19

**Authors:** Yun Wang, Guanyu Wang, Nicolas Moitessier, Anthony K. Mittermaier

**Affiliations:** Department of Chemistry, McGill University, Montreal, QC, Canada

**Keywords:** enzyme catalysis, inhibition, activation, allostery, kinetics, ITC

## Abstract

Isothermal titration calorimetry (ITC) involves accurately measuring the heat that is released or absorbed in real time when one solution is titrated into another. This technique is usually used to measure the thermodynamics of binding reactions. However, there is mounting interest in using it to measure reaction kinetics, particularly enzymatic catalysis. This application of ITC has been steadily growing for the past two decades, and the method is proving to be sensitive, generally applicable, and capable of providing information on enzyme activity that is difficult to obtain using traditional biochemical assays. This review aims to give a broad overview of the use of ITC to measure enzyme kinetics. It describes several different classes of ITC experiment, their strengths and weaknesses, and recent methodological advancements. A summary of applications in the literature is given and several examples where ITC has been used to investigate challenging aspects of enzyme behavior are presented in more detail. These include examples of allostery, where small-molecule binding outside the active site modulates activity. We describe the use of ITC to measure the strength, mode (i.e., competitive, uncompetitive, or mixed), and association and dissociation kinetics of enzyme inhibitors. Further, we provide examples of ITC applied to complex, heterogeneous mixtures, such as insoluble substrates and live cells. These studies exemplify the wide range of problems where ITC can provide answers, and illustrate the versatility of the technique and potential for future development and applications.

## Introduction

Enzymes are catalytic proteins that are ubiquitous in living systems and play central roles in virtually all cellular processes, such as metabolism, active transport, sensing, regulation, communication, and signal transduction and integration (Hunter, 1995; Capaldi and Aggeler, 2002; Benhar et al., 2009; Reyes-Turcu et al., 2009). Consequently enzymes constitute approximately 44% of all validated drug targets, including human enzymes whose dysregulation is linked to disease, and foreign enzymes expressed by pathogens (Zheng et al., 2006). In addition, enzymes are the most efficient catalysts known and have many industrial and medical applications (Choi et al., 2015). For example, hydrolases break polysaccharides down into their component sugars, with applications to food processing, pulp and paper, and biofuel industries (Guzman-Maldonado and Paredes-Lopez, 1995; Sun and Cheng, 2002; Kuhad et al., 2011). Their high selectivity and biocompatibility have also made enzymes useful as therapeutics, for instance in the treatment of phytobezoars (Kramer and Pochapin, 2012).

In general, enzymes show saturation kinetics, which can be rationalized according to the Michaelis–Menten/Briggs–Haldane (MM/BH) model shown in the scheme below

(1) $$E+S\,\overset{{\text{k}}_{1}}{\underset{{\text{k}}_{-1}}{⇌}}E\,S\,\overset{k_{\text{cat}}}{\to }E+P$$

where an enzyme molecule (*E*) binds a substrate (*S*) with association and dissociation rate constants *k*_1_ and *k*_–__1_, respectively, to form the Michaelis complex (ES). The enzyme then acts on the substrate to produce the product (*P*) with a rate constant *k*_cat_. This kinetic scheme gives rise to the familiar MM/BH equation where the enzyme velocity, ν_0_, has a saturable dependence on the substrate concentration:

(2) $$v_{0}=\frac{d\,\left[P\right]}{d\,t}=-\frac{d\,\left[S\right]}{d\,t}=\frac{V_{\text{max}}\,\left[S\right]}{K_{\text{m}}+\left[S\right]}$$

**FIGURE 1:**
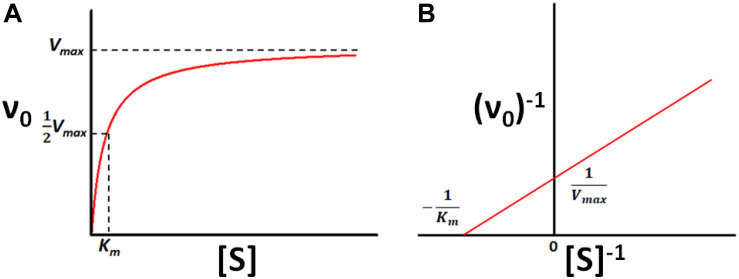
Michaelis-Menten kinetics. **(A)** Velocity (ν_0_) of a typical enzyme-catalyzed reaction versus substrate concentration ([*S*]). **(B)** Double-Reciprocal (Lineweaver–Burk) linearized plot (1/ν_0_ vs. 1/[*S*]) of the rates in **(A)**.

*V*_max_ is the maximum rate of catalysis in the theoretical presence of an infinite quantity of substrate and *K*_m_ is the concentration of substrate required to achieve half-maximal velocity, as illustrated in Figure 1. In terms of the rate constants in Scheme 1,

(3) $$V_{\text{max}}=k_{\text{cat}}\,\left[E\right]$$

and

(4) $$K_{\text{m}}=\frac{k_{-1}+k_{\text{cat}}}{k_{1}}$$

The relationship between enzyme velocity and substrate concentration can be linearized according to the double-reciprocal or Lineweaver–Burk plot, in which ν_0_^–1^ is plotted as a function of [S]^–1^, shown below:

(5) $$\frac{1}{v_{0}}=\frac{K_{\text{m}}}{V_{\text{max}}\,\left[S\right]}+\frac{1}{V_{\text{max}}}$$

The slope of the resulting straight line is *K*_m_/*V*_max_, the *x*-intercept is −*K*_m_^–1^ and the *y*-intercept is *V*_max_^–1^. The parameters *K*_m_ and *k*_cat_ provide simple metrics of an enzyme’s behavior and quantify how activity changes in response to changing solution conditions, addition of inhibitors or activators, changes in the amino acid sequence of the enzyme, chemical modification of the substrate, or exchanging one cofactor for another, among other factors. Thus, methods for measuring *K*_m_ and *k*_cat_ are among the foundational techniques of molecular biosciences.

Most enzyme assays measure the concentrations of substrate and/or product as a function of time. The rates of disappearance and/or appearance give the enzyme velocity, which can be fitted according to Equations 2 or 5. Note that care must be taken in the choice of enzyme and substrate concentrations in order to ensure that both *K*_m_ and *k*_cat_ can be robustly extracted from the data (Stroberg and Schnell, 2016). These experiments can be classified in two types: continuous (or real-time) and discontinuous assays (Harris and Keshwani, 2009). In a continuous assay, the concentrations of substrates or products are measured in the reaction mixture at the same time as catalysis proceeds. For the most part, they employ spectroscopies, such as fluorimetry, UV/vis absorption, or nuclear magnetic resonance, and rely on substrates and products having different spectroscopic signatures (Easterby, 1973; Seethala and Menzel, 1997; Reetz, 2001; Shu and Frieden, 2005). While this is sometimes true in the native reaction, in many cases continuous assays require experimental modifications. Substrates can be chemically altered so that they change color or fluoresce when converted to products (Reetz, 2001). While convenient, this approach has the drawback that a customized substrate must be produced for each enzyme of interest, and non-native chromogenic or fluorogenic substrates do not necessarily have the same reaction kinetics as the natural substrate. Alternatively, in coupled enzyme assays, the reaction mixture includes secondary enzymes that accept the product of the first enzymatic reaction as a substrate and produce downstream spectroscopic changes, such as the interconversion of NAD^+^ and NADH that have very different extinction coefficients for light at 340 nm (Easterby, 1973; McKay and Wright, 1995). This approach allows native substrates to be used, but the assay places limitations on the composition of the reaction mixtures, for example product inhibition or activation studies are impossible (McKay and Wright, 1995) and accurate results depend on choosing appropriate concentrations of the coupled enzymes and secondary substrates. When it is not possible to monitor substrate or product concentrations in real time, discontinuous enzyme assays must be used. In these experiments, the reaction is quenched at various time points after initiation and the substrates and products are separated by an ancillary technique, such as liquid chromatography, gel electrophoresis, centrifugation, or mass spectrometry (Reetz et al., 2004; Hooff et al., 2012; Tauran et al., 2014) and quantified, for instance spectroscopically, radiometrically, or by an immunosorbent assay (Butler, 2000; Kerner and Hoppel, 2002; Hastie et al., 2006). These additional steps add time, expense, and uncertainty to the characterization process.

Isothermal titration calorimetry (ITC) is well known as a powerful tool for studying host/guest binding interactions, but has recently gained in popularity as a general and versatile kinetic assay (Todd and Gomez, 2001; Di Trani et al., 2018a, b). ITC has the advantage of directly measuring the heat flow produced by catalysis in real time (Todd and Gomez, 2001; Di Trani et al., 2017). Since most chemical reactions are either exothermic or endothermic, ITC can be applied to study virtually any enzymatic reaction, without the need for customized reporter molecules, additional coupled enzymes, or post-reaction separation. Furthermore, kinetic ITC experiments can be performed with conventional dilute enzymatic reaction mixtures, even with opaque samples, and require far less enzyme than ITC binding studies (Oezen and Serpersu, 2004). The study of enzyme kinetics has been briefly described in several surveys of the ITC field (Freyer and Lewis, 2008; Liang, 2008; Ghai et al., 2012; Atri et al., 2015) and has been the focus of more technically detailed reviews (Hansen et al., 2016; Mazzei et al., 2016). Here, we discuss how ITC can be applied to a broad array of problems in enzyme biochemistry, including understanding inhibition and allosteric modulation and studying heterogeneous reaction mixtures, from the perspective of our own work in the field. We have tried to choose examples that illustrate how ITC studies can go beyond measuring the parameters usually associated with the term “enzyme kinetics” such as *K*_m_, *k*_cat_, *K*_i_, etc. and extend to observing additional dynamic phenomena like inhibitor association and release, substrates slowly entering the bacterial periplasm, or rearrangements of crystalline chitosan, as described below.

## Enzyme Kinetics by Isothermal Titration Calorimetry

### ITC Instrumentation

**FIGURE 2:**
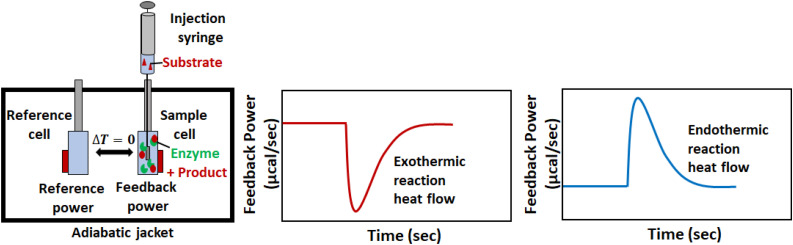
A typical ITC enzyme kinetics experiment. The reaction is initiated when substrate in the syringe is injected into the sample cell containing enzyme. If the reaction is exothermic (endothermic), less (more) feedback power must be supplied to the sample cell to keep it at the same temperature as the reference cell. The instantaneous value of the feedback power is the ITC output.

Isothermal titration calorimetry instruments measure in real time the thermal power that results when one solution (in a syringe) is titrated into another (in a sample cell), as illustrated in Figure 2. A pair of cells, typically coin-shaped or cylindrical with volumes on the order of 200–1,400 μL, are termed the sample and reference cells and contain the analyte solution and reference buffer (or pure water) respectively (Malvern, 2016; TA, 2019). The cells are housed inside a thermostated adiabatic jacket, that is maintained at a temperature slightly below the user-specified value for the cells. Electric resistive heaters, termed the feedback and reference heaters are located on the outer surfaces of the sample and reference cells, respectively, and must supply a constant flow of heat to maintain the cell temperatures at their set point. A Seebeck device sandwiched between the two cells detects any differences in temperature (ΔT) and modulates the power supplied to the feedback heater in order to keep the temperatures of the two cells identical. An automated injection syringe protrudes into the sample cell, which is stirred either by rotation of the paddle-shaped syringe, or by the action of a separate propeller, depending on the make and model of the instrument. A series of injections (typically between 1 and 20 μL) is made into the sample cell. If the reaction between the injectant and analyte is exothermic, there will be a concomitant drop in the power supplied by the feedback heater to maintain a constant temperature. Conversely, if the reaction is endothermic, there will be an increase in feedback power. Once the reaction is complete or the rate becomes negligible, and no further heat is produced or absorbed in the sample cell, the feedback power returns to baseline. The raw output of an ITC instrument is the feedback power measured as a function of time (typically at 1 s intervals). When characterizing binding or reaction thermodynamics, the deflection of the ITC signal from baseline is integrated over the entire injection, and is used to extract enthalpy differences between the unreacted and reacted states (i.e., free vs. bound or substrates vs. products). When characterizing kinetics, the instantaneous output power is interpreted in terms of the reaction velocity, since the rate of heat production or absorption in sample cell is directly proportional to the rate of the reaction. This is slightly complicated by the fact that the ITC signal lags behind heat events in the cell, however, there are several approaches to overcoming this issue, as discussed in later sections. Furthermore, it should be noted that obtaining accurate reaction rates requires accurate heat rates, so it is important to calibrate the calorimetric response (Demarse et al., 2011).

### ITC Kinetics Methods

The instantaneous rate of heat production in the ITC sample cell, dQ/dt, is directly proportional to the reaction velocity (ν_0_ = d[P]/dt) and the enthalpy change of the reaction catalyzed (Δ_r_*H* = *H*_product_ − *H*_substrate_), according to

(6) $$\frac{d\,Q}{d\,t}=V_{\text{cell}}\,\Delta_{\text{r}}\,H\,\frac{d\,\left[P\right]}{d\,t}$$

where *V*_cell_ is the volume of the sample cell. Thus with ITC-derived dQ/dt values obtained as a function of time, it is straightforward to precisely calculate enzyme velocity at any point in the experiment, provided Δ_r_*H* and *V*_cell_ are known. This is obtained from the integrated area of an ITC peak obtained by injecting a known amount of substrate into a sample cell containing sufficient enzyme to rapidly convert it entirely to product,

(7) $$\Delta_{\text{r}}\,H=\frac{\int_{t=0}^{\infty }\frac{d\,Q}{d\,t}\,d\,t}{n_{\text{S}}}$$

where *n*_S_ is the number of moles of substrate injected. In their seminal 2001 paper, Todd and Gomez describe two main approaches for designing ITC experiments that rapidly measure ν_0_ as a function of substrate concentration, allowing the enzyme kinetic parameters to be extracted by fits to Equations 2 or 5. They referred to these as “Pseudo-first Order” and “Continuous” assays, although these terms have been largely replaced with “multiple injection” and “single injection” and we will use the latter terms here. A broad variety of ITC enzyme kinetics experiments have been developed in subsequent years, however, most build on one or the other approach, so it is worthwhile to describe them in some detail, as foundational to the field. In both types of experiment, the reaction is initiated one or more times by mixing enzyme and substrate solutions via injection(s) from the syringe into the sample cell. However, the two methods differ in the concentrations of enzyme and substrate used, the appearance and information content of the data, and the analysis.

#### Multiple Injection Assays

**FIGURE 3:**
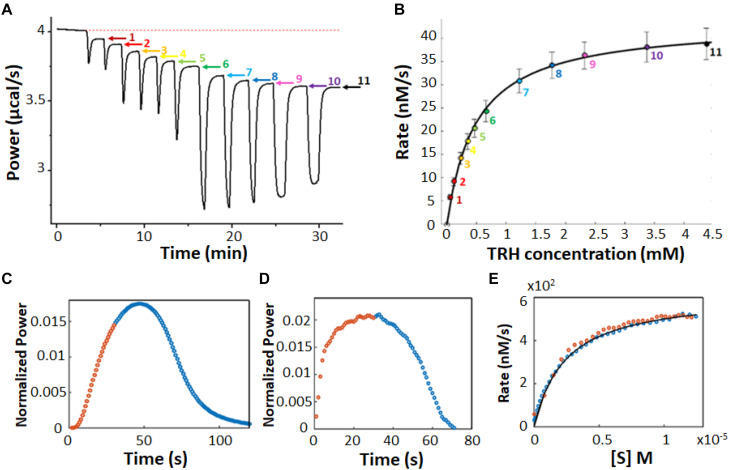
Multiple injection and single injection ITC enzyme kinetic data. **(A)** Multiple injection assay of prolyl oligopeptidase in the sample cell and one of its substrates, thyrotropin releasing hormone, in the syringe (Di Trani et al., 2017). The downward spikes correspond to dilution artifacts from each injection (3, 3, 6, 6, 6, 10, 30, 30, 30, 60, and 60 μL). Larger injections produce larger spikes. The displacement, following each injection, of the horizontal baseline relative to the initial baseline (red dotted line) is proportional to the enzyme velocity. **(B)** MM/BH plot calculated from the data in **(A)**. Error bars correspond to the standard deviations of three repeat experiments. **(C)** Single injection assay with trypsin in the sample cell and one of its substrates, benzoyl-L-arginine ethyl ester, in the syringe (Di Trani et al., 2018b). Data collected during (after) the 30 s injection are plotted in orange (blue). **(D)** Deconvolution of the data in **(C)** to remove the effect of the delayed instrument response according to Equation 10 and an empirical response function. Note that during the first 30 s, the substrate is injected into the sample cell faster than it is consumed and its concentration gradually increases, while the reaction velocity asymptotically approaches the maximum *V*_max_ value, in accordance with the MM/BH Equation (orange circles). After the injection ends, the substrate continues to be consumed and its concentration gradually drops, while the reaction velocity decreases to zero once more (blue circles). **(E)** MM/BH plot generated from the data in **(D)** using Equation 8. The rate versus [*S*] values are superimposable for the injection (orange, increasing [*S*]) and post-injection (blue, decreasing [*S*]) halves of the experiment, providing cross-validation for the data.

In a multiple injection ITC enzyme kinetic assay, the enzyme concentration is chosen to be sufficiently low so that substrate depletion during the experiment is negligible but high enough to provide good signal (Todd and Gomez, 2001). As a result, the instantaneous heat (dQ/dt) and ITC signal are ideally constant (horizontal) between substrate injections and resemble a series of steps, one per injection (Figure 3A). The displacement of each step relative to the initial baseline is directly proportional to ν_0_, according to Equation 6. Exothermic and endothermic reactions give descending and ascending steps, respectively, if the raw feedback power is plotted as a function of time. The injections are designed such that early steps have [*S*] << *K*_m_ and the final injections have nearly saturated the enzyme with [*S*] >> *K*_m_. The concentration of substrate present in the sample cell after each injection is known from the concentration of substrate in syringe and volumes of all injections, while the reaction velocity can be read directly from the vertical position of each step, tracing out a complete Michaelis Menten curve (Figure 3B). In practice, we find that the condition of negligible substrate consumption is met when [*E*] ≤ (10^–4^ s) × *K*_m_/*k*_cat_. Enzyme concentrations that are too high will give steps that slope toward the initial baseline, and will lead to overestimates in the amount of substrate present at each step. Enzyme concentrations that are too low will lead to disappearingly small steps that are obscured by instrument noise.

There are several potential advantages to multiple injection assays compared to single injection ones. Firstly, they can accommodate substantially lower enzyme concentrations. For example in Figures 3A,B, saturation is reached at about 4.5 mM substrate with a *V*_max_ of 40 nM s^–1^. At that rate, it would take more than 10^5^ s or 28 h for the enzyme to convert a sufficient quantity of S to P to complete a single injection assay (see below), which is too long for practical purposes. It should be noted that Δ*_r_H* must be determined in a separate measurement for multiple injection assays, while it is obtained directly from single injection data, thus comparable amounts of enzymes can be consumed when all the necessary experiments are factored in. Secondly, the readout portions of the experiment, i.e., the approximately horizontal signals, are easy to distinguish from injection artifacts, which themselves tend to be smaller since less substrate is added in each injection. Secondly, product accumulation is also less than for single injection assays. In a single injection assay, the amount of product present near the end of an ITC peak is necessarily several-fold greater than the *K*_m_, since the enzyme is initially saturated with substrate. In contrast, much less substrate is converted to product during a multiple-injection experiment, ideally less than 5% (Hansen et al., 2016). Thus much less product is produced during a multiple injection assay compared to a single injection one. This is advantageous when strong product inhibition is present (Wang et al., 2019), although conversely, if product inhibition is of interest yet relatively weak, single injection assays would be the more sensitive option. Furthermore, since the data are drawn from post-injection periods where the enzyme velocities have stabilized to constant values, the timescale of the instrument response to changing heat flow can be ignored, simplifying the analysis (as described below). The main disadvantages are that the determination of a single pair of *k*_cat_ and *K*_m_ values requires a complete series of injections, making this technique relatively slow, and that the total amount of heat generated is much less, making it more susceptible to instrument noise.

#### Single Injection Assays

In a single injection ITC kinetic assay, the amount of enzyme is typically chosen to be large enough so that the injected substrate can be fully converted to product on the timescale of minutes or tens of minutes. The concentration of substrate is chosen so that the injection appreciably saturates the enzyme, i.e., the concentration of substrate in the sample cell immediately after the injection is several-fold higher than the *K*_m_ (Transtrum et al., 2015; Di Trani et al., 2018b). Single injection assays can be initiated either by injecting substrate (syringe) into enzyme (cell) or enzyme (syringe) into substrate (cell). Either case, the ITC feedback power exhibits a large deflection immediately after the injection, decreasing for exothermic reactions and increasing for endothermic ones due to the heat released or absorbed by catalysis. Large heat flows continue as long as the enzyme remains saturated with substrate. The signal gradually returns to the pre-injection baseline as the substrate is consumed as shown in Figure 3C. It should be noted that single injection assays are usually performed with substantially more dilute enzyme, leading to peaks that are much broader, on the order of 20–60 min, in contrast to the 100 s shown here. Our group has been developing ways to collect and analyze data for rapid single injection experiments, such as those shown in Figure 3C, offering over 10-fold reductions in measurement time and opening the door to new types of experiment (Di Trani et al., 2018b; Wang et al., 2019). Single-injection ITC data can be fitted directly by numerically integrating Equation 2 to give [*S*](*t*) and ν_0_(*t*), and calculating dQ/dt as a function of time according to Equation 6, or using non-linear least squares optimization to find the values of *K*_m_ and *k*_cat_ that best reproduce the experimental values. This approach has the advantage that the baseline and instrument response time (see below) can be fitted along with the MM/BH parameters (Transtrum et al., 2015; Di Trani et al., 2017). Alternatively, the concentration of substrate present at any time, *t*, during the heat spike can be calculated by recognizing that the fraction of total substrate remaining at *t* is equal to the ratio of the heat generated after time *t* relative to the total amount of heat generated during the heat spike:

(8) $$\left[S\right]\left(t\right)=\frac{\int_{t}^{\infty }\frac{\text{dQ}}{\text{dt}}\,\text{dt}}{\int_{0}^{\infty }\frac{\text{dQ}}{\text{dt}}\,\text{dt}}\left[S\right]\left(t=0\right)$$

Together, the ν_0_ and [*S*] values trace out a complete Michaelis–Menten curve. We find that substrate is consumed sufficiently rapidly for this technique to be applied when [*E*] ≥ (10^–2^ s) × *K*_m_/*k*_cat_. When the concentration of enzyme is too low, the heat spike persists for such a long time (several hours or more) that the return to baseline is difficult to distinguish. However, the enzyme must be at a low enough concentration so that the return to baseline takes at least seconds to tens of seconds. More rapid reactions start to become obscured by the response function of the instrument (as described below) (Di Trani et al., 2017).

The defining feature of this approach is that a full enzyme kinetic characterization is achieved in a single injection. Thus, with substrate in the syringe, it is straightforward to perform many single injection measurements within the same ITC experiment, simply by programming several injections (as many as 10 or 20) spaced at appropriate intervals (Cai et al., 2001; Ertan et al., 2012; Pedroso et al., 2014; Siddiqui et al., 2014; Maximova and Trylska, 2015; Di Trani et al., 2018b; Mason et al., 2018; Abis et al., 2019; Maximova et al., 2019; Wang et al., 2019). For the sake of clarity, we will refer to these as recurrent single injection experiments, to distinguish them from the very different approach termed multiple injection experiments (see section “Multiple Injection Assays,” above). At the simplest level, recurrent single injection experiments provide repeat measurements of enzyme kinetic parameters and improve the effective signal to noise ratio, although they do not replace true replicate experiments for estimating parameter uncertainties. Catalytic activity is repeatedly characterized over a period of time, giving information on the stability of the enzyme (Mason et al., 2018). These experiments also provide a sensitive measure of product inhibition (or activation), since the product accumulates in the sample cell with each injection (Cai et al., 2001; Wang et al., 2019). The recurrent single injection approach can be adapted to rapidly characterize other types of inhibition as well, as described below (Di Trani et al., 2018b).

Alternatively, single-injection assays can be performed with enzyme in the syringe. This variation is preferable for substrates that are poorly soluble, or those that form suspensions rather than solutions, since they can remain at working (diluted) concentration in the sample cell with constant stirring throughout the experiment (Lonhienne et al., 2001; Ali et al., 2013a, b; Commin et al., 2013; Pedroso et al., 2014; Lehoczki et al., 2016; Kaeswurm et al., 2019). Similarly, if very high substrate concentrations (100 s of mM) are needed (e.g., for enzymes with very large *K*_m_ values), it can be unfeasible to inject sufficient amounts of substrate without generating large injection heat artifacts, related to the large dilutions. Instead, the concentrated substrate can be equilibrated in the sample cell and small injections of dilute enzyme can be used to initiate the reaction (Lonhienne and Winzor, 2002; Lonhienne et al., 2003). Lastly, the barrel of the injection syringe lies exterior to ITC insulated jacket. If experiments are being performed at temperatures approaching the enzyme melting point, placing the enzyme in the syringe (which is at ambient temperature) allows it to spend as little time as possible at high and destabilizing temperatures (Ali et al., 2013a, b, 2015). It is worth noting that with enzyme in the syringe, recurrent single injection assays, as described above, are not possible, since the maximum concentration of substrate is necessarily present at the beginning of the measurement and cannot be replenished once conversion to product is complete. Another consideration is that small amounts of material can leak from the tip of the injection syringe during the long initial equilibration step, as well as between injections (although these delays are shorter). While this is true for both substrate- and enzyme-injection setups, the leakage is potentially far more serious with enzyme in the syringe, as this can act on the substrate in sample cell throughout the equilibration period, consuming much or all of it before the experiment has begun. In contrast, leakage of a few μL of substrate from the tip of the syringe does not dramatically imperil the procedure. Consequently, it is recommended to employ a buffer “plug” when injecting enzyme, a few μL of buffer that is drawn up into the needle after loading the syringe with an enzyme solution (Malvern, 2010, Malvern, 2014).

#### Rapid Enzyme Kinetics Measured by ITC

In many cases, the ITC signal can be considered nearly equal (and technically opposite) to the instantaneous rate of heat generation in the sample cell (i.e., ≈−dQ/dT). This approximation holds when the relevant portions of the heat signal vary slowly with time, such as in multiple injection assays and in cases where the peaks for single injection assays are broad (tens of minutes). For short reactions with rapidly varying heat signals, the situation becomes substantially more complicated. There are several physical processes that must occur before the heat generated by enzymatic catalysis is detected in the ITC output (Todd and Gomez, 2001; Burnouf et al., 2012). These include a heat transfer delay, which is the length of time necessary for the solid phase thermocouple to detect the small change in sample cell temperature (Wiseman et al., 1989; Freire et al., 1990; García-Fuentes et al., 1998; Ebrahimi et al., 2015) and the electronic response that alters the power supplied to the feedback heater, driving the temperature gradient between the cells back to zero (Wiseman et al., 1989). These steps are typically described collectively as an instrument response function, *f*(*t*) which can be thought of as the instrument signal that would result from an instantaneous burst of heat being released in the sample cell. If the release of heat in the sample cell is described by the time-dependent function *h*(*t*), then the instrument output is given by

(9) $$g\,\left(t\right)=f\,\left(t\right)⊗h\,\left(t\right)=\int_{0}^{t}f\,\left(\tau \right)\,g\,\left(t-\tau \right)\,d\,\tau$$

where ⊗ indicates the convolution. The finite instrument response has the effect of spreading out the observed signal compared to the actual heat profile, such that peaks begin more gradually and die away more slowly. The instrument response function is often assumed to have a simple exponential shape (López-Mayorga et al., 1987; García-Fuentes et al., 1998; Velázquez-Campoy et al., 1999; Burnouf et al., 2012; Vander Meulen and Butcher, 2012) $f\,\left(t\right)\propto e\,x\,p\,\left\{-\frac{t}{\tau }\right\}$, where τ is referred to as the response time and is typically on the order of 5–15 s (Burnouf et al., 2012). Accounting for the instrument response can be done in one of two ways. In the first, non-linear least squares fitting can be used to find the enzyme kinetic parameters that generate an instantaneous heat function, *h*(*t*), which when convoluted with the assumed instrument response function, *f*(*t*), best reproduces the ITC peak shape, *g*(*t*). In the second, one can use the Tian equation and the assumed value of τ to mathematically remove the spreading effect of the instrument response (Backman et al., 1994).

We have recently shown that the assumption of a simple exponential response function is incompatible with experimental ITC peak shapes, and that *f*(*t*) is a more complicated function of time. We found that the response function can instead be equated to the signal obtained from very short (0.1 s) injections of a model host/guest system, such as EDTA injected with Ca^2+^. We termed this approach an empirical response model (ERM), and it reproduces ITC peaks quantitatively, producing sub-second time resolution (Di Trani et al., 2017). The empirical response model can be used for direct fitting to raw ITC data, according to Equation 9, or can be used to deconvolute the instantaneous heat function from the instrument response (Di Trani et al., 2017, 2018b). This second approach relies on the convolution theorem which states that, given Equation 9, then

(10) ℱ⁢(g⁢(t))=ℱ⁢(f⁢(t))⋅ℱ⁢(h⁢(t))

where ℱ indicates the Fourier transform. The deconvoluted instantaneous heat function is then given by $h\,\left(t\right)={ℱ}^{-1}\,\left(\frac{ℱ\,\left(g\,\left(t\right)\right)}{ℱ\,\left(f\,\left(t\right)\right)}\right)$, as exemplified in Figures 3C–E. It must be emphasized that the instrument response [*f*(*t*)] varies with the manufacturer and model, as well as the temperature, solution viscosity, and stirring speed, among other factors, and must be measured using very short injections (e.g., Ca^2+^/EDTA) performed under conditions as close to those of the experiment of interest as possible (Di Trani et al., 2017). This approach is only really necessary when measuring reactions that take place on the same timescale as the instrument response (i.e., roughly less than 20–30 s). For slow reactions that take tens of minutes or more to complete, the instrument response can be largely ignored, while for intermediate timescale reactions, the approximation of a single instrument response time, τ, is adequate.

An alternative approach, termed initial rate calorimetry (IrCal), avoids the issue of modeling the instrument response function altogether (Honarmand Ebrahimi et al., 2015). Honarmand Ebrahimi et al. (2015) found empirically that the initial slope of the ITC signal is proportional to the peak velocity of the enzyme after the injection. The constant of proportionality can be determined by calibration experiments. A series of substrate injections of different sizes is made, and the initial slopes of the injections reveal how the ν_0_ varies with [*S*]. One drawback of this approach is that each injection gives only a single ν_0_ value, in contrast to a typical ITC single injection assay, which yields tens to hundreds of ν_0_ values at different [*S*].

### ITC Enzyme Kinetics Applications

#### Overview

We have performed a comprehensive search of the scientific literature and identified 73 publications between 2001 and 2019 reporting ITC-derived kinetic data on 59 different enzymes including hydrolases, transferases, oxidoreductases, lyases, ligases, and a protein folding chaperonin, listed in Supplementary Table 1. The authors explained their choice of ITC with a variety of reasons, including that ITC can represent the only continuous assay available, that it can exploit the native substrate where alternative continuous assays require chemically-modified chromogenic or fluorogenic substrates, that ITC avoids potential artifacts associated with coupled enzyme assays, and that ITC allows continuous assays to be performed on heterogeneous and spectroscopically opaque mixtures. multiple injection-type ITC experiments were used for 35 enzymes, single injection-type ITC experiments were used for 27 enzymes, and enzyme-injection assays were used for 8 enzymes. Several of these publications focused on the development of new ITC kinetics approaches, such as IrCal and ERM above, and others are described below. Many of these studies focused on characterizing homogeneous enzymes exhibiting classical MM/BH kinetics. However, many others described more complex systems, such as enzymes with cooperative kinetics, those interacting with allosteric effectors or inhibitors, and those in heterogeneous media, such as insoluble hydrated polymers or even living cells. We describe some interesting examples from our own work and the work of others below.

#### Allostery and Cooperativity

Allostery is a key feature of biological systems in which covalent modification or ligand binding at one site influences the activity at distant sites in a macromolecule or macromolecular assembly. Allosteric regulation plays a central role in metabolism and cell signaling (Guarnera and Berezovsky, 2019) and has been identified as a source of new drug targets (Monod et al., 1965; Koshland et al., 1966; Perutz, 1989; DeDecker, 2000; Fenton, 2008; Guarnera and Berezovsky, 2020); thus, detailed descriptions of allostery have far-reaching implications (Zhang et al., 2020). For example, the downstream products of a biosynthetic pathway can down-regulate the activity of the enzyme catalyzing the first committed step, maintaining balance between different branches of core metabolism through the process of feedback inhibition (Spencer and Raffa, 2004; Gerhart, 2014). Alternatively, enzymes may require allosteric activators in order to function, providing an extra layer of control (Willemoës and Sigurskjold, 2002; Lunn et al., 2008). In the special case that the substrate itself acts as an allosteric effector, enzyme kinetics necessarily deviate from the classical MM/BH model. This can often be accounted for mathematically with a Hill coefficient of cooperativity, *n*, such that the enzyme velocity is given by the expression

(11) $$v_{0}=\frac{V_{\text{max}}\,{\left[S\right]}^{n}}{K_{\text{m}}^{\text{n}}+{\left[S\right]}^{n}}$$

Values of *n* > 1 indicate positive cooperativity, such that substrate binding makes an enzyme more active toward additional substrates, and give characteristically sigmoidal ν_0_ vs. [*S*] plots. In a simple interpretation, an enzyme with a given Hill coefficient, *n*, either binds exactly *n* molecules of substrate or none at all. When binding a molecule of substrate at an allosteric site reduces enzyme activity toward additional substrates (substrate inhibition), enzyme velocity can often be described by the expression

(12) $$\frac{d\,\left[P\right]}{\text{dt}}=\frac{V_{\text{max}}\,\left[S\right]+V_{\text{max}}^{{}^{\prime }}\,\left(\frac{{\left[S\right]}^{2}}{K_{\text{i}}^{{}^{\prime }}}\right)}{K_{\text{m}}+\left[S\right]\,\left(1+\frac{K_{\text{m}}}{K_{\text{i}}}\right)+\left(\frac{{\left[S\right]}^{2}}{K_{\text{i}}^{{}^{\prime }}}\right)}$$

where *V*_max_ is the maximum velocity of the reaction when the allosteric site is empty, *V*′_max_ is the maximum velocity when the allosteric site is filled, and *K*_i_ and *K*′_i_ are the equilibrium dissociation constants for substrate binding at the allosteric site when the active site is empty and filled, respectively (Jeoh et al., 2005).

**FIGURE 4:**
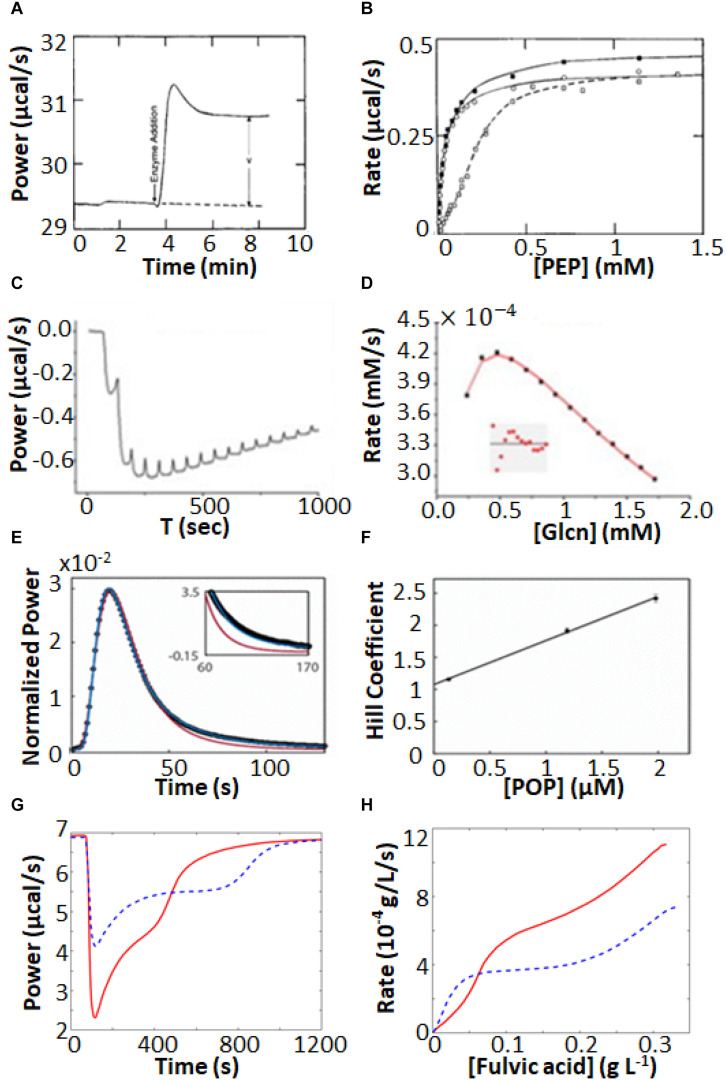
Non-MM/BH enzyme kinetics observed by ITC. **(A)** Single injection experiments with pyruvate kinase in the syringe and phosphoenolpyruvate and ADP in the sample cell (Lonhienne and Winzor, 2002). The displacement of the horizontal baseline is proportional to the velocity of the enzyme. **(B)** Baseline displacements (ΔP) obtained at different [PEP] (O), in the presence of phenylalanine as an allosteric effector (□), and in the presence of phenylalanine and proline as a molecular crowding agent (■). **(C)** Multiple injection assay with gluconokinase and ATP in the sample cell and gluconate in the syringe (Rohatgi et al., 2015). **(D)** Enzyme velocities from **(C)**, fitted to a variant of Equation 12 that accounts for formation of the non-productive E⋅ADP⋅gluconate ternary complex. **(E)** Single injection assay with substrate (thyrotropin releasing hormone) in the syringe and prolyl oligopeptidase (POP) in the sample cell (Di Trani et al., 2017). Points are experimental data, red and blue curves are the best fits with classical MM/BH model, and cooperative model (Equation 11) with *n* = 2.4, respectively. **(F)** Dependence of the extracted Hill coefficient on POP concentration (0.125, 1.2, and 2 μM). **(G)** Single injection assay with versatile peroxidase in the sample cell and fulvic acid in the syringe, exhibiting biphasic cooperative kinetics (Siddiqui et al., 2014). **(H)** Reaction rates as s function of substrate concentration calculated from **(G)**. In **(G,H)**, data were extracted from the original reference using Graph Grabber v2.0.2 (Quintessa) and plotted using MATLAB (MathWorks); red solid curves indicate the first injection and blue dashed curves indicate the second injection.

Isothermal titration calorimetry represents a powerful tool for characterizing complex enzyme allosteric interactions. For instance, ITC was used to measure the kinetics of pyruvate kinase (PK) (Lonhienne and Winzor, 2002; Lonhienne et al., 2003) which catalyzes the transfer of a phosphate from phosphoenolpyruvate to ADP as part of the last step of glycolysis. Allosteric binding of the amino acid phenylalanine (Phe) shifts PK to an inactive form, and is believed to be related to cellular damage in the genetic disease phenylketonuria (Hörster et al., 2006; van Spronsen et al., 2009). Lonhienne and Winzor (2002), Lonhienne et al. (2003) were interested in how the presence of osmolytes affected the active/inactive transition. PK has traditionally been studied using a coupled enzyme assay, which is suboptimal for studying the effects of high concentrations of osmolytes, since it is challenging to distinguish effects on PK from effects on the secondary enzymes of the coupled assay. ITC avoids these issues, since enzyme activity is detected directly. They performed enzyme injection assays (Figure 4A), where the displacement in the baseline after enzyme is injected is proportional to the velocity of the reaction. While this approach avoids large injection artifacts, it is somewhat time consuming as separate experiment must be performed for each data point in Figure 4B. They obtained standard MM/BH curves in the absence of Phe. In the presence of 6 mM Phe, the curve shifts to right, indicating a lower substrate affinity, and develops sigmoidal character, a hallmark of positive cooperativity. Interestingly, addition of the osmolyte proline shifted the curve back to the original location, corresponding to a return of the inactive state to the active state.

In another example, Rohatgi et al. (2015) used ITC to fully characterize the complex kinetic mechanism of gluconokinase, which transfers a phosphate from ATP to the common nutrient gluconate. They used multiple injection enzyme assays, injecting gluconate into enzyme at a constant ATP concentration. The traces clearly show declining activity at higher substrate concentrations indicative of substrate inhibition (Figures 4C,D). Interestingly the shapes of the plots varied as a function of ATP concentration in a way that was consistent with substrate inhibition occurring via the formation of an enzyme⋅ADP⋅gluconate ternary complex.

Our lab recently used ITC to characterize prolyl-oligopeptidase (POP) a validated drug target for multiple myeloma (Di Trani et al., 2018a) allowing study of the native peptide substrate, rather than the chemically-modified colorigenic substrate analog that is typically used. We performed ITC single injection assays and found that at lower enzyme concentrations, data were well fit by the standard MM/BH equation, while at higher concentrations, cooperativity became more apparent, with *n* > 2 at an enzyme concentration of 2 μM (Figures 4E,F). At the high enzyme concentrations where this behavior becomes apparent, the reactions go to completion in 10 s or less, underlining the potential of ITC to characterize rapid reaction kinetics.

More exotic ITC thermograms were obtained for the versatile peroxidase (VP) from *Bjerkandera adusta*, which has potential applications in the degradation of the industrial and agricultural materials (Abdel-Hamid et al., 2013; Wang et al., 2013). VP can employ both lignin peroxidase and manganese peroxidase mechanisms in the degradation of humic materials (Siddiqui et al., 2014). ITC single injection assays (fulvic acid injected into VP) performed under conditions where both mechanisms are active gave biphasic heat spikes where each phase can be attributed to one of the mechanisms and each showed cooperative kinetics (Figures 4G,H). The data were well fit by the modified Hill Equation

(13) $$v=\frac{V_{\mathrm{max1}}\,{\left[S\right]}^{n\,1}}{K_{\mathrm{m1}}^{\mathrm{n1}}+{\left[S\right]}^{n\,1}}+\frac{V_{\mathrm{max2}}\,{\left[S\right]}^{n\,2}}{K_{\mathrm{m2}}^{\mathrm{n2}}+{\left[S\right]}^{n\,2}}$$

with *n*1 = 1.7 for the 1st phase and *n*2 = 10 for the 2nd phase. Interestingly, the second injection gave broader peaks than the first, indicative of product inhibition, as described in more detail below.

#### Enzyme Inhibitors

Quantitative information on inhibitor binding is critical for developing drugs (Su and Xu, 2018) and understanding how enzymes function in living systems (Hulme and Trevethick, 2010). In fact ITC is primarily used to measure these types of host–guest interactions and a quick literature search for “ITC and inhibitor” yields more than 1,300 articles (using Clarivate Analytics Web of Science). In a traditional ITC experiment, the enzyme and inhibitor are placed in the sample cell and injection syringe, respectively, and a series of injections are made, while the instrument records the heat released or absorbed by the binding process itself (Su and Xu, 2018). This gives a wealth of thermodynamic information on the interaction, since the Gibbs energy and enthalpy of binding are detected separately, as the shape and overall magnitude of the saturation isotherm, respectively (Sigurskjold, 2000; Tellinghuisen, 2008; Keller et al., 2012; Su and Xu, 2018). Under favorable circumstances, the kinetics of binding can be measured as well (Burnouf et al., 2012; Piñeiro et al., 2019).

However, there are some drawbacks to this approach. Firstly, traditional ITC experiments require substantially more material than many other techniques used to measure binding, such as fluorescence or surface plasmon resonance. The recommended concentration of enzyme in the sample cell is roughly 5–500 times the inhibitor dissociation constant, *K*_i_, [i.e., Wiseman “c” values of 5–500 (Wiseman et al., 1989)] often leading to requirements for protein on the micromolar to tens of micromolar scale (Malvern, 2010, Malvern, 2014). On the flip side, enzyme/inhibitor interactions that are too tight can be challenging since the enzyme concentration should not be more than about 1,000-fold greater than *K*_i_, and very low enzyme concentrations lead to vanishingly small heat signals. This can be overcome with competition assays (Velazquez-Campoy and Freire, 2006), but the procedure is far more complicated. Finally, a traditional ITC binding experiment is not suited to characterizing all modes of inhibition. Competitive inhibitors bind exclusively to the free enzyme (E) and are suitable for traditional ITC binding experiments. In contrast, uncompetitive inhibitors bind exclusively to the enzyme/substrate Michaelis complex (ES), and in principle would not show an interaction at all in a traditional experiment. Mixed inhibitors bind to both E and ES, but the results of a traditional experiment would not reflect the true inhibition properties of the compound. Furthermore, an ITC binding experiment alone does not contain sufficient information to identify the inhibition mode.

These drawbacks can be overcome with ITC-based enzyme kinetic experiments. Firstly ITC kinetics experiments require far less enzyme than binding experiments. In a binding experiment, a single molecule of enzyme generates heat only once, when it forms a complex with the inhibitor. Whereas in a kinetics experiment, a single molecule of enzyme produces heat continuously as it undergoes multiple turnover. This allows ITC enzyme kinetics experiments to routinely be performed with sub-nM protein concentrations, which is outside the typical concentration range of ITC binding experiments. Furthermore ITC kinetics experiments are suitable for all modes of inhibition and can be performed in such a way that the mode and associated parameters are clearly evident. Finally, as detailed below, ITC differs fundamentally from other enzyme assays in that it detects the instantaneous velocity directly, while other methods measure concentrations of substrates, products, or reporters as a function of time and extract enzyme velocity indirectly. This makes ITC uniquely sensitive to how enzyme velocity changes with time, for instance as inhibitors exert their influence. Thus ITC has great potential for measurement of inhibitor association and dissociation rates.

A quantitative analysis of enzyme inhibition typically involves determination of the mode (competitive, uncompetitive, or mixed) and the inhibitor dissociation constant *K*_i_. For mixed-mode inhibitors, there are separate *K*_i_ values for binding to E and to ES. Apparent *K*_m_^app^ and *k*_cat_^app^ values are measured at different concentrations of inhibitor [I] and analyzed collectively to extract the inhibition parameters. For a competitive inhibitor

(14) $$k_{\text{cat}}^{\text{app}}=k_{\text{cat}};K_{\text{m}}^{\text{app}}=K_{\text{m}}\,\left(1+\frac{\left[I\right]}{K_{\text{i}}}\right)$$

and a double-reciprocal plot of 1/ν_0_ vs. 1/[*S*] obtained at different [*I*] gives a series of lines that intersect at the *y*-axis. For an uncompetitive inhibitor

(15) $$k_{\text{cat}}^{\text{app}}=\frac{k_{\text{cat}}}{1+\frac{\left[I\right]}{K_{\text{i}}^{\prime }}};K_{\text{m}}^{\text{app}}=\frac{K_{\text{m}}}{1+\frac{\left[I\right]}{K_{\text{i}}^{\prime }}}$$

where *K*′_i_ is the dissociation constant for the inhibitor and ES complex and a double-reciprocal plot gives a series of parallel lines. For mixed inhibitors

(16) $$k_{\text{cat}}^{\text{app}}=\frac{k_{\text{cat}}}{1+\frac{\left[I\right]}{K_{\text{i}}^{\prime }}};K_{\text{m}}^{\text{app}}=K_{\text{m}}\,\frac{\left(1+\frac{\left[I\right]}{K_{\text{i}}}\right)}{\left(1+\frac{\left[I\right]}{K_{\text{i}}^{\prime }}\right)}$$

and a double-reciprocal plot gives a series of lines that intersect elsewhere than the *y*-axis. In the case that *K*_i_ = *K*′_i_, the inhibitor is said to be non-competitive and the lines intersect at the *x*-axis.

**FIGURE 5:**
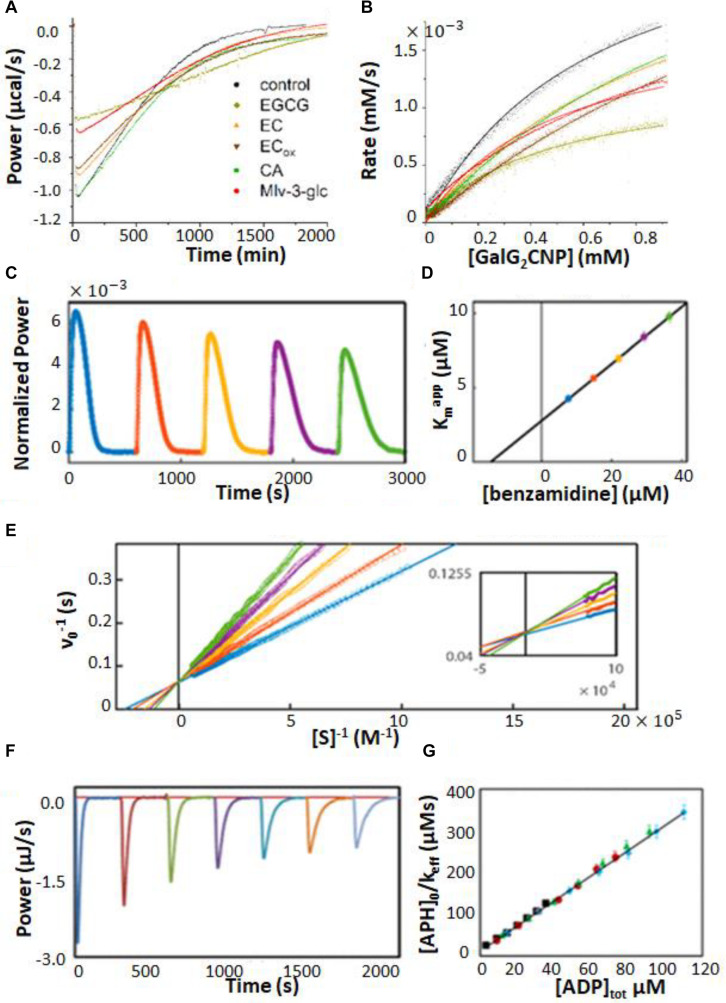
Enzyme inhibition characterized by ITC single injection-type assays. **(A)** Inverse injection assay with α-amylase in the syringe and the substrate 2-chloro-4-nitrophenyl-maltoside (GalG_2_CNP) in the syringe together with a variety of inhibitors: ACA (acarbose), CA (chlorogenic acid), EC (epicatechin), ECox (oxidized epicatechin), EGCG (epigallocatechin gallate), Mlv-3-glc (malvidin-3-glucoside) (Hanhineva et al., 2010). **(B)** MM/BH curves calculated from the curves in **(A)**. **(C)** Single injection assay with substrate (benzoyl-L-arginine ethyl ester) and inhibitor (benzamidine) in the syringe and trypsin in the sample cell (Di Trani et al., 2018b). **(D)** *K*_m_^app^ values extracted from direct fits to each of the injections (different colors) in **(C)**. **(E)** Data from **(C)**, deconvoluted using the empirical response model (Equation 10), converted to ν_0_ and [*S*] and presented as a double-reciprocal plot. **(F)** Single injection assay with substrate (ATP) in the syringe and aminoglycoside-3′-phosphotransferase IIIa (APH) and kanamycin A in the sample cell (Wang et al., 2019). Under these dilute conditions [ATP] << *K*_m_, ITC peaks decay exponentially with rate constant *k*_eff_ = *k*_cat_/*K*_m_. *k*_eff_ decreases with each injection due to product inhibition by ADP. **(G)** Plot of [APH]/*k*_eff_ as a function of total accumulated ADP concentration.

Characterization of enzyme inhibition can largely be accomplished with the experiments described in Section “ITC Kinetics Methods.” For example, ITC was used to characterize inhibitors of pancreatic α-amylase, which hydrolyses starches into monosaccharides in the gut (Kaeswurm et al., 2019). It has been proposed that a variety of polyphenol plant metabolites inhibit α-amylase, slowing glucose absorption by the intestine, and reducing spikes in insulin levels with implications for the management of diabetes (Hanhineva et al., 2010). The authors tested a panel of naturally occurring polyphenols, together with the known potent α-amylase inhibitor acarbose. They used a single injection assay where α-amylase was injected into 1 mM trisaccharide substrate, with or without 100 μM of each phenolic inhibitor. The enzyme injection technique allowed them to initiate the reaction with very small (1 μL) additions of dilute reagent (1 μM enzyme), thereby almost entirely avoiding injection artifacts. Each experiment consists of a single peak, corresponding to the complete conversion of substrate to product over the course of about 2.5 h. The resulting ITC isotherms are shown in Figure 5A and MM/BH plots calculated from these data are shown in Figure 5B. Interestingly, all of the polyphenols affected both the *k*_cat_ of the enzyme (indicated by the height of the asymptote in Figure 5B) and the *K*_m_, indicating mixed modes of inhibition, although the curves were not fitted quantitatively in this study. Mixed inhibition was also observed using experiments with a colorimetric assay detected by UV/vis spectroscopy. Note that while these assays were performed injecting enzyme into substrate pre-incubated with inhibitor, similar assays can also be performed by injecting substrate into enzyme pre-incubated with inhibitors (Catucci et al., 2019).

When ITC inhibition experiments are performed with the inhibitor loaded in the sample cell prior to data collection, as in the examples above, then the procedure must be repeated several times in order to accurately measure inhibition parameters. This demands a considerable investment of time, since the cleaning, loading, equilibration, and data collection must be performed separately for each inhibitor concentration. Our lab has developed a procedure for considerably shortening this timeline, allowing much higher throughput of samples (Di Trani et al., 2018b). This approach is a variation of a standard recurrent single injection assay, with the modification that the syringe contains both substrate and inhibitor. A series of injections is made with each ITC peak giving a *k*_cat_ and *K*_m_ pair. The inhibitor accumulates with each injection, such that the activity of the enzyme decreases in each successive peak, and provides a thorough sampling of different inhibitor concentrations. Typical data are shown in Figure 5C, with each peak clearly broader than the preceding one, reflecting slower catalysis by the enzyme (trypsin inhibited with benzamidine in this case). Each peak is complete within 5–10 min and a total data set can be collected in under an hour. The peaks were analyzed by fitting directly to the raw ITC data. The extracted *k*_cat_^app^ was very similar for each peak, while the *K*_m_^app^ increased linearly with increasing peak number and inhibitor concentration (Figure 5D), as expected for a competitive inhibitor (Equation 14). The y-intercept of the line is *K*_m_ and the slope is *K*_m_/*K*_i_. Alternatively, the peaks can be deconvoluted using the empirical response function (see Rapid Enzyme Kinetics Measured by ITC, above) and converted to double-reciprocal plots in a model-free manner (Figure 5E). The data give a series of straight lines that intersect at the *y*-axis, consistent with the competitive inhibition mechanism of benzamidine. This approach gives full kinetic characterization for as many as ten different inhibitor concentrations in a single experiment, providing assessment of inhibition mode and strength. Note that direct fitting of ITC peaks is more straightforward and is generally preferred to deconvolution-based approaches. The double-reciprocal plot is primarily useful for illustrating the extent to which data follow the MM/BH equation and obey a given pattern of inhibition, as it is easier to spot deviations from linearity and identify intersections than it is to judge the shapes of ITC peaks by eye. More recently, this method was applied to the study of human soluble epoxide hydrolase, which is involved in cardiovascular homeostasis, hypertension, nociception, and insulin sensitivity through the metabolism of a variety of epoxy-fatty acids (Abis et al., 2019). There is interest in inhibiting this enzyme in order to raise physiological levels of epoxy-fatty acids, which have been shown to have beneficial biological activities, and to reduce the levels of the reaction products, dihydroxy fatty acids, many of which are cytotoxic (Wagner et al., 2017). Previous assays of this enzyme relied on liquid chromatography/tandem mass spectrometry which is a discontinuous method with non-negligible liquid handling steps. The authors showed that single injection ITC assays can clearly distinguish the different kinetics of different epoxy fatty acids providing a simpler and more robust route to characterization. As well, they validated the multiple inhibitor injection method for this system using a previously identified antagonist, setting the stage for rapid ITC-based screening for inhibitors of this enzyme.

A similar type of situation occurs when the enzyme is inhibited by the reaction product. In this case, the product of the reaction accumulates after each injection, leading to progressively slower catalysis. In fact, slowing catalysis with subsequent injections in a single injection ITC experiment is a hallmark of product inhibition (Cai et al., 2001). A challenge can arise when inhibition is strong, since the product generated by a single injection can be sufficient to essentially abrogate activity of the enzyme in subsequent injections. This was the case in our studies of several bacterial kinases and their inhibition by the reaction product ADP (Wang et al., 2019). Interestingly, ADP inhibition of kinases cannot be studied using the standard coupled enzyme assay for kinase activity, since ADP is reconverted to ATP by the secondary enzymes (McKay and Wright, 1995). In order to avoid excessive inhibition of the enzymes, we injected the substrate ATP at a concentration well below the *K*_m_. Under these conditions, substrate consumption follows simple first-order kinetics with rate constant, *k*_eff_ = *k*_cat_[*E*]/*K*_m_^app^, where *K*_m_^app^ increases with each injection as product (i.e., inhibitor) accumulates. Data for dilute ATP injected into aminoglycoside-3′-phosphotransferase IIIa are shown in Figure 5F, clearly exhibiting broadening of successive peaks. A plot of [*E*]/*k*_eff_ vs. [*I*] is linear (Figure 5G) with a slope of $\frac{K_{\text{m}}}{k_{\text{cat}}}\cdot \frac{1}{K_{\text{i}}}$ and a y-intercept of $\frac{K_{\text{m}}}{k_{\text{cat}}}$; the ratio of the two gives the inhibition constant, *K*_i_. Using this approach, we found that the *K*_i_ for ADP was comparable or lower than the *K*_m_ for ATP, for all three kinases studied, implying that inhibition by ADP influences kinase activity *in vivo*. We also identified a more complex mechanism for a dimeric pantothenate kinase, wherein ADP is activating at low concentrations and becomes inhibitory at higher concentrations, consistent with allosteric communication between the two active sites (Wang et al., 2019).

**FIGURE 6:**
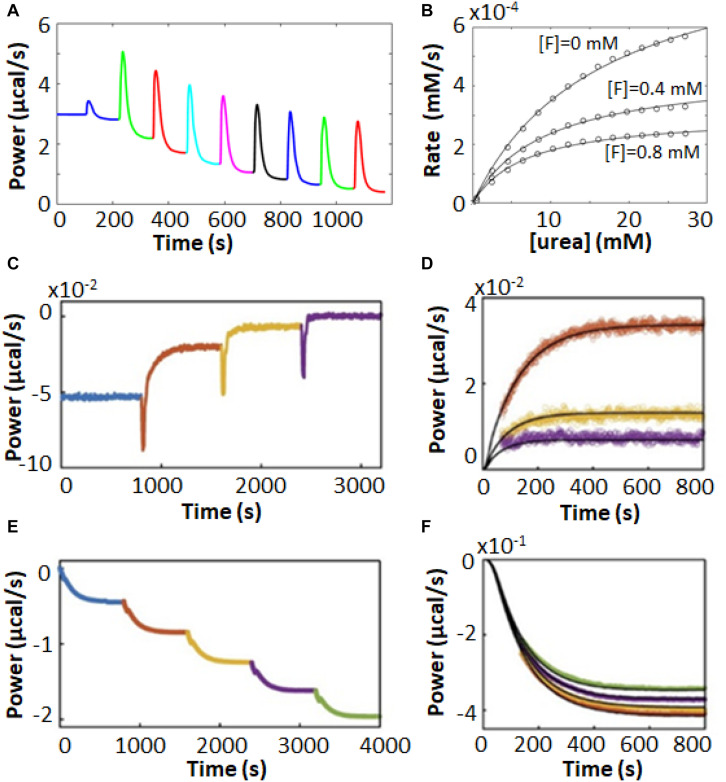
Enzyme inhibition characterized by ITC Pseudo-First-Order-type assays. **(A)** Multiple injection-type ITC assays with urea in the syringe and urease in the sample cell (Benini et al., 2014). **(B)** MM/BH plots from data similar to **(A)** with fluoride ion concentrations of 0, 0.4, and 0.8 mM, fitted to a mixed inhibition model (Equation 16). In **(A,B)**, data were extracted from the original reference using Graph Grabber v2.0.2 (Quintessa) and plotted using MATLAB (MathWorks). **(C)** Inhibitor association kinetics experiment with prolyl oligopeptidase (POP) and substrate (thyrotropin releasing hormone, TRH) in the sample cell and reversible covalent inhibitor in the syringe (Di Trani et al., 2018a). **(D)** Overlay of injections 1–3 from **(C)** (colored points) with best global fits to a kinetic model of association (black curves). **(E)** Inhibitor dissociation kinetics experiment with TRH in the sample cell and POP and a reversible covalent inhibitor in the syringe (Di Trani et al., 2018a). **(F)** Overlay of injections 2–5 from **(E)** (colored points) with fit best global fits to a kinetic model of dissociation (black curves).

As discussed in Section “Multiple Injection Assays,” there are some advantages associated with multiple injection ITC enzyme assays, and this holds true for inhibitor characterization as well. For instance, the urease enzyme acts on urea to produce bicarbonate and two equivalents of ammonia. Multiple injection ITC assays produce far less reaction product than do single injection ones; in this case it helps to minimize the production of ammonia which is alkaline, volatile, and corrosive. Urease inhibitors have potential antimicrobial and agricultural applications (Kosikowska and Berlicki, 2011; Upadhyay, 2012). Benini et al. (2014) used multiple injection ITC assays to characterize inhibition of urease by fluoride ions. Typical raw ITC data are shown in Figure 6A, while the corresponding MM/BH curves for urease activity in the presence of 0, 400, and 800 μM NaF are shown in Figure 6B, revealing a mixed mode of inhibition. Interestingly, the competitive and uncompetitive components (*K*_i_ and *K*′_i_ in Equation 16) showed different pH dependencies, and the authors could link the two different inhibition modes to two different locations of fluoride ion binding in the active site, as determined by X-ray crystallography.

Our lab has recently designed a pair of experiments which build on the multiple injection ITC experiment to give additional information on inhibitor association and dissociation rates (Di Trani et al., 2018a). In the association experiment (Figure 6C), the syringe contains the inhibitor and the sample cell contains dilute enzyme and sufficient substrate to maintain an essentially constant concentration throughout the experiment. The rate of catalysis is initially constant, giving a horizontal line. A series of injections is made, in this case of reversible covalent inhibitors targeting prolyl oligopeptidase in the sample cell. In each case, the enzyme was increasingly inhibited and the power values shifted upward, since the rate of (exothermic) catalysis was reduced after each injection. As highlighted in Figure 6D, this shift occurred gradually over tens to hundreds of seconds, which corresponds to the time required for the inhibitor to bind in the active site. Furthermore, the upward shift of the ITC signal became smaller for each subsequent injection, as the enzyme became increasingly saturated with inhibitor. The decrease in the sizes of the steps is related to the inhibition constant, *K*_i_, and the values of *k*_on_ and *K*_i_ can be fitted numerically to the data. The disassociation rate can then be calculated as *k*_off_ = *k*_on_ × *K*_i_.

In the dissociation experiments, the sample cell contains only the substrate and the syringe contains enzyme saturated with an inhibitor (prolyl oligopeptidase and a reversible covalent inhibitor), which is added to the cell in a series of injections (Figure 6E). Immediately following each injection there was no change in the rate of catalysis in the sample cell as the added enzyme was fully inhibited. However, the large dilution (>20-fold) experienced by the injectant led to a net dissociation of the inhibitor and a gradual downward shift of the ITC signal as the freshly released enzyme began to act on the substrate (Figure 6F). The downward shift of the ITC signal became smaller for each subsequent injection, as the inhibitor accumulated in the sample cell and the net dissociation of each injection diminished. The decrease in the sizes of the steps is governed by the value of *K*_i_. Data for the series of injections can be fitted simultaneously to yield *k*_off_ and *K*_i_ (Figure 6F). The association rate can then be calculated as *k*_on_ = *k*_off_/*K*_i_. Note that concentrations of enzyme are so low in these experiments (≈10 nM) that ITC detects only heats of catalysis, while heats of inhibitor/enzyme binding can be safely ignored. This method exploits the fact that ITC measures enzyme velocity directly. A traditional concentration-based enzyme assay would detect the gradual decreases and increases in enzyme velocity vividly illustrated in Figures 6D,F as slight curvature in the product buildup curve, making quantitative analysis far more difficult (Di Trani et al., 2018a).

#### Heterogeneous Mixtures

**FIGURE 7:**
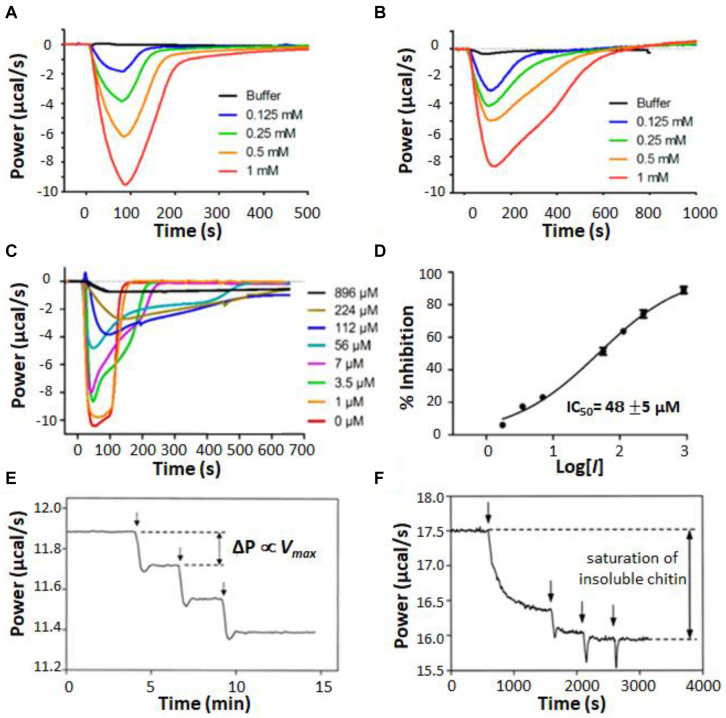
Isothermal titration calorimetry characterization of heterogeneous mixtures. **(A)** Single injection assays with substrate (cefazolin) in the syringe and purified NDM-1 in the sample cell (Zhang et al., 2018). **(B)** Single injection assays with cefazolin in the syringe and a suspension of live *E. coli* bacteria expressing NDM-1 in the sample cell. **(C)** Experiments in **(B)** repeated with various concentrations of an inhibitor (D-captopril) added to the *E. coli* suspension. **(D)** IC_50_ calculation, taking the magnitude of each peak in **(C)** as proportional to enzyme activity. Single injection assays with chitinase in the injection syringe and **(E)** soluble chitin fragments or **(F)** insoluble chitin in the sample cell (Lonhienne et al., 2001). Vertical arrows indicate timings of injections.

A unique aspect of ITC enzyme kinetic assays is their general ability to provide real-time measurements on opaque systems that are unsuitable for typical bulk spectroscopic techniques. One example of this is ITC enzyme kinetics experiments performed on suspensions of living cells (Wang et al., 2017, 2018; Zhang et al., 2018; Lv et al., 2019). Comparing the behavior of enzymes *in vitro* and *in situ* is critical for understanding how they work in living systems and can reveal how enzyme kinetics are tied to additional layers of biological dynamics. Furthermore, studying enzymes in the intact organism avoids the question of whether activity has been compromised by extraction and circumvents the need for purification steps at all. For instance, Zhang et al. (2018) used ITC to study the metallo-β-lactamase NDM-1 in living cultures of *Escherichia coli*. NDM-1 cleaves carbapenems, providing bacterial resistance to these “last resort” β-lactam antibiotics. Development of NDM-1 inhibitors has the potential to resensitize resistant bacteria and offers an avenue for treating these kinds of serious drug-resistant infections (Zhang et al., 2018). NDM-1 is located in the periplasm of Gram-negative bacteria, anchored to the inner leaflet of the outer membrane by a lipidated cysteine residue (Palzkill, 2013). Thus the natural environment of NDM-1 is not well recapitulated by purified enzymes in solution. The authors used cefazolin as a test substrate, injecting it into either purified NDM-1 (Figure 7A) or live *E. coli* bacteria expressing the enzyme (Figure 7B). Very similar single injection-type heat signals were obtained in both cases, with all injected substrate being consumed within 100–200 s in the case of purified protein and 400–600 s in the case of live cells. Cefazolin injected in live bacterial cultures not expressing NDM-1 gave negligible heat signals. The amount of NDM-1 present in the live cells was not determined and differences in enzyme concentration could have contributed to differences in kinetic profiles in Figures 7A,B. However, the authors also made the intriguing suggestion that the slower kinetics in live cells could be due, at least in part, to the time lag of cefazolin entering the periplasm and hydrolyzed products leaving. The results presented in their study suggest that ITC could be a powerful tool for studying these processes in future work. The authors went on to test a live-cell screen for NDM-1 inhibitors, using a panel of previously reported compounds. Data are shown in Figure 7C for a similar assay to Figure 7B, with cefazolin injected in live cells pre-incubated with different concentrations of known inhibitor, D-captopril; as the concentration of inhibitor was raised, the ITC heat peaks became increasingly broad, indicative of slower catalysis and enzyme inhibition. Taking the absolute amplitudes of the peaks as a measure of enzyme activity, the set of ITC data gave an IC_50_ of about 50 μM (Figure 7D), in good agreement with previous measurements on purified protein (Zhang et al., 2018). The authors repeated the experiments on four different clinical strains of pathogenic bacteria. Interestingly, different strains showed different levels of activity, perhaps reflecting different levels of NDM-1 expression, different accessibility of the periplasmic space, or both. This study paves the way for using ITC both as a rapid screen for inhibitors of antibiotic resistance genes, and also as a tool for probing the resistance profiles of clinical isolates.

Other examples of opaque reaction mixtures are those involving insoluble substrates (Lonhienne et al., 2001; Murphy et al., 2010a, b, 2012, 2013) or enzymes immobilized on insoluble matrices (Henzler et al., 2008; Henao-Escobar et al., 2014; Ali et al., 2015; Mason et al., 2018) where the components are combined as a suspension or slurry. These mixtures are of great industrial importance, in part because the insoluble carbohydrate polymers cellulose and chitin are the two most abundant organic compounds on earth, present in large quantities in vascular plants and arthropod exoskeletons, respectively (Berlemont et al., 2016). Cellulose and chitin modifying enzymes have many potential applications in biofuel production, chemical upcycling, agriculture, and textile production (Turner et al., 2007). Lonhienne et al. (2001) used ITC to characterize the activity of psychrophilic bacterial chitinases, which hydrolyse glycosidic bonds in chitin. They studied both an insoluble suspension of powdered chitin and a sample in which the chitin had been cleaved by acid hydrolysis into soluble fragments of 40–100 kDa. When a series of injections of dilute chitinase were made into a sample of soluble chitin fragments, the heat flow increased in a series of steps of equal size (Figure 7E), as expected, since for normal MM/BH kinetics, the total reaction velocity is proportional to the concentration of enzyme. In contrast, when chitinase was injected into the chitin suspension, the first injection produced a large increase in heat flow, the step associated with the second injection was much smaller, and subsequent injections did not increase the rate of catalysis at all (Figure 7F). The authors attributed this to saturation of the chitin substrate. The bulk of the material forms an interconnected network buried in the colloidal particle and is therefore inaccessible to enzymatic attack. Once the surfaces of the particles are covered by enzyme molecules, the addition of further enzyme does not increase the rate of hydrolysis. An additional interesting feature of the ITC data is the slow and gradual increase in enzyme velocity after the first injection. The steady-state rate of catalysis was not reached until roughly 15 min after the enzyme was added. The authors attribute this lag to the opening of crystalline regions on the colloid surface to expose new fibril ends that are susceptible to the enzyme. This study vividly illustrates how ITC data can give information not only on enzyme kinetics, but also on the dynamic processes to which the enzyme activity is linked.

## Discussion

The methods and examples discussed above illustrate the power and potential of ITC as a universal enzyme assay. ITC offers real-time monitoring of enzymatic reactions in cases where other types of continuous assays are unavailable. This is exemplified by human soluble epoxide hydrolase (Abis et al., 2019) discussed above, where previous work had relied on a combination of quenching the reaction at various time points and analyzing the composition by liquid chromatography and tandem mass spectrometry (Morisseau et al., 2010). The ability to employ natural substrates is another large asset for ITC. This is particularly true when the MM/BH parameters obtained for chemically modified colorigenic or fluorogenic substrate analogs do not match those obtained for the native substrate by ITC. For example, glycosidase activity can be measured spectrophotometrically with synthetic substrates, such as maltooligomer derivatives with chromogenic chloro-nitrophenyl (CNP) groups attached (Klaus et al., 1999; Morishita et al., 2000; Ramasubbu et al., 2005). Separate studies on α-amylase and glycogen phosphorylase found the *K*_m_ values of the fluorogenic substrate analogs to be substantially lower than those of the native substrates determined by ITC, possibly due to interactions of the chromophore with the active site of the enzyme (Lehoczki et al., 2016; Szabó et al., 2019). Thus ITC represents a simple way to accurately characterize how enzymes interact with their biologically relevant molecular partners. ITC also offers advantages for enzymes where the standard assay involves indirect readout with a coupled-enzyme system. This is particularly true when adding co-solutes or inhibitors that affect enzymatic activity since the secondary enzymes can be affected as well as the enzyme of interest, as discussed above for pyruvate kinase (Lonhienne and Winzor, 2002; Lonhienne et al., 2003). In addition, testing spectroscopically active inhibitors or other effector molecules can become a challenge when using spectrophotometric assays, i.e., with chromogenic or fluorogenic probes, or with coupled assays. In contrast, deeply-colored inhibitors are fully compatible with ITC inhibition assays (Zebisch et al., 2012). Furthermore ITC’s ability to characterize opaque samples further extends the reach of this technique beyond spectroscopically-accessible systems. The examples described above involving suspensions of live cells (Wang et al., 2017, 2018; Zhang et al., 2018; Lv et al., 2019) and insoluble substrates (Lonhienne et al., 2001; Murphy et al., 2010a, b, 2012, 2013), illustrate how the surrounding milieu can influence enzyme activity and how ITC can be a probe of these more complex dynamics.

Over the past 20 years, the number of publications using ITC to measure enzyme kinetics has been growing at an ever-accelerating rate (Supplementary Figure 1). The advantages of ITC described above are becoming increasingly recognized, and we expect that this trend will continue as the technique becomes more visible and widely known. Most of the studies to date have employed the two main types of experiment described in the original paper by Todd and Gomez (2001), i.e., multiple injection and single injection assays. However, we believe that the full potential of ITC as an enzyme kinetic technique is only starting to be explored and that the development of innovative methods and novel capabilities will help to drive the further growth of the user community. Our group (Di Trani et al., 2017, 2018a, 2018b; Wang et al., 2019) and others (Honarmand Ebrahimi et al., 2015; Transtrum et al., 2015) have recently reported methodological advances that push limits of the technique. One area of interest is the development of strategies for quantitatively addressing the finite response time of ITC instruments (Honarmand Ebrahimi et al., 2015; Transtrum et al., 2015; Di Trani et al., 2017). For example, our Empirical Response Model, described above, has performed well under a variety of conditions, and allowed us to, for example, characterize very rapid reactions and clearly identify non-MM/BH kinetics from single ITC peaks (Di Trani et al., 2017). In developing this model, we found that the kinetics of post-injection mixing can be largely ignored under many conditions. However, this approximation should likely be revisited and is an area ripe for advancement, perhaps by incorporating some ideas used in analyzing binding kinetics by ITC (Burnouf et al., 2012). More generally, there are an enormous number of ways to generate different ITC experiments. As an illustration, for a simple ternary system of enzyme/substrate/inhibitor, one can imagine that each of the components can be loaded in either the syringe or the sample cell, and can be either dilute (*C* << *K*_m_, *K*_i_) or concentrated (C >> *K*_m_, *K*_i_). This, in principle, gives 64 distinct arrangements, only a few of which have been investigated to date. There are likely scenarios in which many of these hypothetical experiments would yield uniquely useful data. When one considers the number of multi-substrate enzymes (Wang et al., 2019) and multiple enzyme systems with shared substrates or products (Murphy et al., 2010a), as well as the effects of allostery and substrate or product inhibition, the complexity of the experiment-space and versatility of ITC starts to become apparent. Finally, the ability of ITC to extract meaningful kinetic data from systems as complicated as biopolymer suspensions (Henzler et al., 2008; Ali et al., 2015) or even living cells (Wang et al., 2017, 2018; Zhang et al., 2018; Lv et al., 2019) holds great promise for understanding enzyme behavior *in situ* and *in vivo*. One could imagine expanding this approach to a multitude of other complex and heterogeneous media, such as purified cellular components, homogenized tissue or soil samples, and nanostructured materials, to name a few. It is our belief that between advancements in experimental design and analysis and sample selection and preparation, the full potential of ITC to study enzyme kinetics will become evident in the coming years.

## Author Contributions

YW, GW, NM, and AM wrote the article. All authors contributed to the article and approved the submitted version.

## Conflict of Interest

The authors declare that the research was conducted in the absence of any commercial or financial relationships that could be construed as a potential conflict of interest.

## References

[B1] Abdel-Hamid A. M. Solbiati J. O. Cann I. K. O. (2013). “Insights into lignin degradation and its potential industrial applications,” in *Advances in Applied Microbiology*, Vol. 82 eds Sariaslani S. Gadd G. M. (Cambridge, MA: Academic Press), 1–28. 10.1016/b978-0-12-407679-2.00001-6 23415151

[B2] Abis G. Pacheco-Gómez R. Bui T. T. T. Conte M. R. (2019). Isothermal titration calorimetry enables rapid characterization of enzyme kinetics and inhibition for the human soluble epoxide hydrolase. *Anal. Chem.* 91 14865–14872. 10.1021/acs.analchem.9b01847 31660733 PMC7041903

[B3] Ali G. Dulong V. Gasmi S. N. Rihouey C. Picton L. Le Cerf D. (2015). Covalent immobilization of pullulanase on alginate and study of its hydrolysis of pullulan. *Biotechnol. Progr.* 31 883–889. 10.1002/btpr.2093 25919860

[B4] Ali G. Rihouey C. Larreta-Garde V. Le Cerf D. Picton L. (2013a). Molecular size characterization and kinetics studies on hydrolysis of pullulan by pullulanase in an entangled alginate medium. *Biomacromolecules* 14 2234–2241. 10.1021/bm400371r 23713899

[B5] Ali G. Rihouey C. Le Cerf D. Picton L. (2013b). Effect of carboxymethyl groups on degradation of modified pullulan by pullulanase from *Klebsiella pneumoniae*. *Carbohydr. Polym.* 93 109–115. 10.1016/j.carbpol.2012.07.039 23465908

[B6] Atri M. S. Saboury A. A. Ahmad F. (2015). Biological applications of isothermal titration calorimetry. *Phys. Chem. Res.* 3 319–330.

[B7] Backman P. Bastos M. Hallen D. Lonnbro P. Wadso I. (1994). Heat conduction calorimeters: time constants, sensitivity and fast titration experiments. *J. Biochem. Biophys. Methods* 28 85–100. 10.1016/0165-022x(94)90023-x 8040566

[B8] Benhar M. Forrester M. T. Stamler J. S. (2009). Protein denitrosylation: Enzymatic mechanisms and cellular functions. *Nat. Rev. Mol. Cell Biol.* 10 721–732. 10.1038/nrm2764 19738628

[B9] Benini S. Cianci M. Mazzei L. Ciurli S. (2014). Fluoride inhibition of Sporosarcina pasteurii urease: structure and thermodynamics. *J. Biol. Inorg. Chem.* 19 1243–1261. 10.1007/s00775-014-1182-x 25113581

[B10] Berlemont R. Martiny A. C. Martiny A. C. (2016). Glycoside hydrolases across environmental microbial communities. *PLoS Comput. Biol.* 12:e1005300. 10.1371/journal.pcbi.1005300 27992426 PMC5218504

[B11] Burnouf D. Ennifar E. Guedich S. Puffer B. Hoffmann G. Bec G. (2012). kinITC: a new method for obtaining joint thermodynamic and kinetic data by isothermal titration calorimetry. *J. Am. Chem. Soc.* 134 559–565. 10.1021/ja209057d 22126339

[B12] Butler J. E. (2000). Enzyme-Linked immunosorbent assay. *J. Immun.* 21 165–209. 10.1080/01971520009349533 10929886

[B13] Cai L. Cao A. Lai L. (2001). An isothermal titration calorimetric method to determine the kinetic parameters of enzyme catalytic reaction by employing the product inhibition as probe. *Anal. Biochem.* 299 19–23. 10.1006/abio.2001.5397 11726179

[B14] Capaldi R. A. Aggeler R. (2002). Mechanism of the F1F0-type ATP synthase, a biological rotary motor. *Trends Biochem. Sci.* 27 154–160. 10.1016/s0968-0004(01)02051-5 11893513

[B15] Catucci G. Sadeghi S. J. Gilardi G. (2019). A direct time-based ITC approach for substrate turnover measurements demonstrated on human FMO3. *Chem. Commun.* 55 6217–6220. 10.1039/c9cc01356c 31074479

[B16] Choi J.-M. Han S.-S. Kim H.-S. (2015). Industrial applications of enzyme biocatalysis: current status and future aspects. *Biotechnol. Adv.* 33 1443–1454. 10.1016/j.biotechadv.2015.02.014 25747291

[B17] Commin C. Aumont-Nicaise M. Claisse G. Feller G. Da Lage J.-L. (2013). Enzymatic characterization of recombinant α-amylase in the *Drosophila melanogaster* species subgroup: is there an effect of specialization on digestive enzyme? *Genes Genet. Syst.* 88 251–259. 10.1266/ggs.88.251 24463528

[B18] DeDecker B. S. (2000). Allosteric drugs: thinking outside the active-site box. *Chem. Biol.* 7 R103–R107. 10801477 10.1016/s1074-5521(00)00115-0

[B19] Demarse N. A. Quinn C. F. Eggett D. L. Russell D. J. Hansen L. D. (2011). Calibration of nanowatt isothermal titration calorimeters with overflow reaction vessels. *Anal. Biochem.* 417 247–255. 10.1016/j.ab.2011.06.014 21741951

[B20] Di Trani J. M. De Cesco S. O’Leary R. Plescia J. do Nascimento C. J. Moitessier N. (2018a). Rapid measurement of inhibitor binding kinetics by isothermal titration calorimetry. *Nat. Commun.* 9:893. 10.1038/s41467-018-03263-3 PMC5832847 29497037

[B21] Di Trani J. M. Moitessier N. Mittermaier A. K. (2017). Measuring rapid time-scale reaction kinetics using isothermal titration calorimetry. *Anal. Chem.* 89 7022–7030. 10.1021/acs.analchem.7b00693 28590118

[B22] Di Trani J. M. Moitessier N. Mittermaier A. K. (2018b). Complete kinetic characterization of enzyme inhibition in a single isothermal titration calorimetric experiment. *Anal. Chem.* 90 8430–8435. 10.1021/acs.analchem.8b00993 29926719

[B23] Easterby J. S. (1973). Coupled enzyme assays: a general expression for the transient. *Biochim. Biophys. Acta* 293 552–558. 10.1016/0005-2744(73)90362-8 4711820

[B24] Ebrahimi K. Hagedoorn P.-L. Jacobs D. Hagen W. R. (2015). Accurate label-free reaction kinetics determination using initial rate heat measurements. *Sci. Rep.* 5:16380. 10.1038/srep16380 PMC4647221 26574737

[B25] Ertan H. Siddiqui K. S. Muenchhoff J. Charlton T. Cavicchioli R. (2012). Kinetic and thermodynamic characterization of the functional properties of a hybrid versatile peroxidase using isothermal titration calorimetry: Insight into manganese peroxidase activation and lignin peroxidase inhibition. *Biochimie* 94 1221–1231. 10.1016/j.biochi.2012.02.012 22586704

[B26] Fenton A. W. (2008). Allostery: an illustrated definition for the ‘second secret of life’. *Trends Biochem. Sci.* 33 420–425. 10.1016/j.tibs.2008.05.009 18706817 PMC2574622

[B27] Freire E. Mayorga O. L. Straume M. (1990). Isothermal titration calorimetry. *Anal. Chem.* 62 950A–959A.

[B28] Freyer M. W. Lewis E. A. (2008). Methods in cell biology. *Acad. Press.* 84 79–113. 10.1016/S0091-679X(07)84004-0 17964929

[B29] García-Fuentes L. Barón C. Mayorga O. L. (1998). Influence of dynamic power compensation in an isothermal titration microcalorimeter. *Anal. Chem.* 70 4615–4623. 10.1021/ac980203u 9823721

[B30] Gerhart J. (2014). From feedback inhibition to allostery: the enduring example of aspartate transcarbamoylase. *FEBS J.* 281 612–620. 10.1111/febs.12483 23953008

[B31] Ghai R. Falconer R. J. Collins B. M. (2012). Applications of isothermal titration calorimetry in pure and applied research-survey of the literature from 2010. *J. Mol. Recognit.* 25 32–52. 10.1002/jmr.1167 22213449

[B32] Guarnera E. Berezovsky I. N. (2019). On the perturbation nature of allostery: sites, mutations, and signal modulation. *Curr. Opin. Struct. Biol.* 56 18–27. 10.1016/j.sbi.2018.10.008 30439587

[B33] Guarnera E. Berezovsky I. N. (2020). Allosteric drugs and mutations: chances, challenges, and necessity. *Curr. Opin. Struct. Biol.* 62 149–157. 10.1016/j.sbi.2020.01.010 32062398

[B34] Guzman-Maldonado H. Paredes-Lopez O. (1995). Amylolytic enzymes and products derived from starch: a review. *Crit. Rev. Food Sci. Nutr.* 35 373–403. 10.1080/10408399509527706 8573280

[B35] Hanhineva K. Torronen R. Bondia-Pons I. Pekkinen J. Kolehmainen M. Mykkanen H. (2010). Impact of dietary polyphenols on carbohydrate metabolism. *Int. J. Mol. Sci.* 11 1365–1402. 10.3390/ijms11041365 20480025 PMC2871121

[B36] Hansen L. D. Transtrum M. K. Quinn C. Demarse N. (2016). Enzyme-catalyzed and binding reaction kinetics determined by titration calorimetry. *Biochim. Biophys. Acta Gen. Subj.* 1860 957–966. 10.1016/j.bbagen.2015.12.018 26721335

[B37] Harris T. K. Keshwani M. M. (2009). *Methods Enzymology*, Vol. 463 Cambridge, MA: Academic Press, 57–71. 10.1016/S0076-6879(09)63007-X 19892167

[B38] Hastie C. J. McLauchlan H. J. Cohen P. (2006). Assay of protein kinases using radiolabeled ATP: a protocol. *Nat. Protoc.* 1 968–971. 10.1038/nprot.2006.149 17406331

[B39] Henao-Escobar W. Domínguez-Renedo O. Alonso-Lomillo M. A. Cascalheira J. Dias-Cabral A. Arcos-Martínez M. (2014). Characterization of a disposable electrochemical biosensor based on putrescine oxidase from micrococcus rubens for the determination of putrescine. *Electroanalysis* 27 368–377. 10.1002/elan.201400387

[B40] Henzler K. Haupt B. Ballauff M. (2008). Enzymatic activity of immobilized enzyme determined by isothermal titration calorimetry. *Anal. Biochem.* 378 184–189. 10.1016/j.ab.2008.04.011 18440294

[B41] Honarmand Ebrahimi K. Hagedoorn P.-L. Jacobs D. Hagen W. R. (2015). Accurate label-free reaction kinetics determination using initial rate heat measurements. *Sci. Rep.* 5:16380. 10.1038/srep16380 PMC4647221 26574737

[B42] Hooff G. P. van Kampen J. J. A. Meesters R. J. W. van Belkum A. Goessens W. H. F. Luider T. M. (2012). Characterization of β-Lactamase enzyme activity in bacterial lysates using MALDI-mass spectrometry. *J. Prot. Res.* 11 79–84. 10.1021/pr200858r 22013912

[B43] Hörster F. Schwab M. A. Sauer S. W. Pietz J. Hoffmann G. F. Okun J. G. (2006). Phenylalanine reduces synaptic density in mixed cortical cultures from mice. *Pediatr. Res.* 59 544–548. 10.1203/01.pdr.0000203091.45988.8d 16549526

[B44] Hulme E. C. Trevethick M. A. (2010). Ligand binding assays at equilibrium: validation and interpretation. *Br. J. Pharmacol.* 161 1219–1237. 10.1111/j.1476-5381.2009.00604.x 20132208 PMC3000649

[B45] Hunter T. (1995). Protein kinases and phosphatases: the Yin and Yang of protein phosphorylation and signaling. *Cell* 80 225–236. 10.1016/0092-8674(95)90405-0 7834742

[B46] Jeoh T. Baker J. O. Ali M. K. Himmel M. E. Adney W. S. (2005). β-d-Glucosidase reaction kinetics from isothermal titration microcalorimetry. *Anal. Biochem.* 347 244–253. 10.1016/j.ab.2005.09.031 16269126

[B47] Kaeswurm J. A. H. Claasen B. Fischer M.-P. Buchweitz M. (2019). Interaction of structurally diverse phenolic compounds with porcine pancreatic α-Amylase. *J. Agric. Food Chem.* 67 11108–11118. 10.1021/acs.jafc.9b04798 31496243

[B48] Keller S. Vargas C. Zhao H. Piszczek G. Brautigam C. A. Schuck P. (2012). High-precision isothermal titration calorimetry with automated peak-shape analysis. *Anal. Chem.* 84 5066–5073. 10.1021/ac3007522 22530732 PMC3389189

[B49] Kerner J. Hoppel C. L. (2002). Radiochemical malonyl-CoA decarboxylase assay: Activity and subcellular distribution in heart and skeletal muscle. *Anal. Biochem.* 306 283–289. 10.1006/abio.2002.5696 12123667

[B50] Klaus L. Barbara G. Florian R. (1999). Evaluation of a direct α-amylase assay using 2-Chloro-4-nitrophenyl-α-D-maltotrioside. *Clin. Chem. Lab. Med.* 37 1053–1062. 10726812 10.1515/CCLM.1999.154

[B51] Koshland, D. E. Jr Nemethy G. Filmer D. (1966). Comparison of experimental binding data and theoretical models in proteins containing subunits. *Biochemistry* 5 365–385. 10.1021/bi00865a047 5938952

[B52] Kosikowska P. Berlicki L. (2011). Urease inhibitors as potential drugs for gastric and urinary tract infections: a patent review. *Exp. Opin. Ther. Pat.* 21 945–957. 10.1517/13543776.2011.574615 21457123

[B53] Kramer S. J. Pochapin M. B. (2012). Gastric phytobezoar dissolution with ingestion of diet coke and cellulase. *Gastroenterol. Hepatol.* 8 770–772. PMC3966177 24672417

[B54] Kuhad R. C. Gupta R. Singh A. (2011). Microbial cellulases and their industrial applications. *Enzyme Res.* 280696:280610. 10.4061/2011/280696 PMC3168787 21912738

[B55] Lehoczki G. Szabó K. Takács I. Kandra L. Gyémánt G. (2016). Simple ITC method for activity and inhibition studies on human salivary α-amylase. *J. Enzyme Inhibit. Med. Chem.* 31 1648–1653. 10.3109/14756366.2016.1161619 27052104

[B56] Liang Y. (2008). Applications of isothermal titration calorimetry in protein science. *Acta Biochim. Biophys. Sin.* 40 565–576. 10.1111/j.1745-7270.2008.00437.x 18604448

[B57] Lonhienne T. Baise E. Feller G. Bouriotis V. Gerday C. (2001). Enzyme activity determination on macromolecular substrates by isothermal titration calorimetry: application to mesophilic and psychrophilic chitinases. *Biochim. Biophys. Acta* 1545 349–356. 10.1016/s0167-4838(00)00296-x 11342059

[B58] Lonhienne T. G. A. Reilly P. E. B. Winzor D. J. (2003). Further evidence for the reliance of catalysis by rabbit muscle pyruvate kinase upon isomerization of the ternary complex between enzyme and products. *Biophys. Chem.* 104 189–198. 10.1016/s0301-4622(02)00366-6 12834837

[B59] Lonhienne T. G. A. Winzor D. J. (2002). Calorimetric demonstration of the potential of molecular crowding to emulate the effect of an allosteric activator on pyruvate kinase kinetics. *Biochemistry* 41 6897–6901. 10.1021/bi020064h 12033921

[B60] López-Mayorga O. Mateo P. L. Cortijo M. (1987). The use of different input signals for dynamic characterisation in isothermal microcalorimetry. *Sci. Instr.* 20:265 10.1088/0022-3735/20/3/006

[B61] Lunn F. A. MacDonnell J. E. Bearne S. L. (2008). Structural requirements for the activation of *Escherichia coli* CTP synthase by the allosteric effector GTP are stringent, but requirements for inhibition are lax. *J. Biol. Chem.* 283 2010–2020. 10.1074/jbc.m707803200 18003612

[B62] Lv M. Zhang Y.-J. Zhou F. Ge Y. Zhao M.-H. Liu Y. (2019). Real-time monitoring of D-Ala-D-Ala dipeptidase activity of VanX in living bacteria by isothermal titration calorimetry. *Anal. Biochem.* 578 29–35. 10.1016/j.ab.2019.05.002 31071297

[B63] Malvern (2010). *VP-ITC Microcalorimeter User’s Manual.* Cambridge: Malvern.

[B64] Malvern (2014). *ITC-200 Microcalorimeter User’s Manual.* Cambridge: Malvern.

[B65] Malvern (2016). *Microcal itc Systems:Understanding Biomolecular Interactions.* Cambridge: Malvern.

[B66] Mason M. Scampicchio M. Quinn C. F. Transtrum M. K. Baker N. Hansen L. D. (2018). Calorimetric methods for measuring stability and reusability of membrane immobilized enzymes. *J. Food Sci.* 83 326–331. 10.1111/1750-3841.14023 29278666

[B67] Maximova K. Trylska J. (2015). Kinetics of trypsin-catalyzed hydrolysis determined by isothermal titration calorimetry. *Anal. Biochem.* 486 24–34. 10.1016/j.ab.2015.06.027 26119333

[B68] Maximova K. Wojtczak J. Trylska J. (2019). Enzyme kinetics in crowded solutions from isothermal titration calorimetry. *Anal. Biochem.* 567 96–105. 10.1016/j.ab.2018.11.006 30439369

[B69] Mazzei L. Ciurli S. Zambelli B. (2016). Methods enzymol. *Elsevier* 567 215–236. 10.1016/s0962-8924(00)89005-4 26794356

[B70] McKay G. A. Wright G. D. (1995). Kinetic mechanism of aminoglycoside phosphotransferase type IIIa. Evidence for a Theorell-Chance mechanism. *J. Biol. Chem.* 270 24686–24692. 10.1074/jbc.270.42.24686 7559583

[B71] Monod J. Wyman J. Changeux J.-P. (1965). On the nature of allosteric transitions: a plausible model. *J. Mol. Biol.* 12 88–118. 10.1016/s0022-2836(65)80285-6 14343300

[B72] Morishita Y. Iinuma Y. Nakashima N. Majima K. Mizuguchi K. Kawamura Y. (2000). Total and pancreatic amylase measured with 2-chloro-4-nitrophenyl-4-O-β-D-galactopyranosylmaltoside. *Clin. Chem.* 46 928–933. 10.1093/clinchem/46.7.928 10894835

[B73] Morisseau C. Inceoglu B. Schmelzer K. Tsai H. J. Jinks S. L. Hegedus C. M. (2010). Naturally occurring monoepoxides of eicosapentaenoic acid and docosahexaenoic acid are bioactive antihyperalgesic lipids. *J. Lipid Res.* 51 3481–3490. 10.1194/jlr.m006007 20664072 PMC2975720

[B74] Murphy L. Baumann M. J. Borch K. Sweeney M. Westh P. (2010a). An enzymatic signal amplification system for calorimetric studies of cellobiohydrolases. *Anal. Biochem.* 404 140–148. 10.1016/j.ab.2010.04.020 20457121

[B75] Murphy L. Bohlin C. Baumann M. J. Olsen S. N. Sørensen T. H. Anderson L. (2013). Product inhibition of five Hypocrea jecorina cellulases. *Enzyme Microb. Technol.* 52 163–169. 10.1016/j.enzmictec.2013.01.002 23410927

[B76] Murphy L. Borch K. McFarland K. C. Bohlin C. Westh P. (2010b). A calorimetric assay for enzymatic saccharification of biomass. *Enzyme Microb. Technol.* 46 141–146. 10.1016/j.enzmictec.2009.09.009

[B77] Murphy L. Cruys-Bagger N. Damgaard H. D. Baumann M. J. Olsen S. N. Borch K. (2012). Origin of initial burst in activity for Trichoderma reesei endo-glucanases hydrolyzing insoluble cellulose. *J. Biol. Chem.* 287 1252–1260. 10.1074/jbc.m111.276485 22110134 PMC3256860

[B78] Oezen C. Serpersu E. H. (2004). Thermodynamics of aminoglycoside binding to Aminoglycoside-3’-phosphotransferase IIIa studied by isothermal titration calorimetry. *Biochemistry* 43 14667–14675. 10.1021/bi0487286 15544337

[B79] Palzkill T. (2013). Metallo-β-lactamase structure and function. *Ann. N.Y. Acad. Sci.* 1277 91–104. 10.1111/j.1749-6632.2012.06796.x 23163348 PMC3970115

[B80] Pedroso M. M. Ely F. Lonhienne T. Gahan L. R. Ollis D. L. Guddat L. W. (2014). Determination of the catalytic activity of binuclear metallohydrolases using isothermal titration calorimetry. *Eur. J. Biochem.* 19 389–398. 10.1007/s00775-013-1079-0 24414447

[B81] Perutz M. F. (1989). Mechanisms of cooperativity and allosteric regulation in proteins. *Quart. Rev. Biophys.* 22 139–237. 10.1017/s0033583500003826 2675171

[B82] Piñeiro Á Muñoz E. Sabín J. Costas M. Bastos M. Velázquez-Campoy A. (2019). AFFINImeter: a software to analyze molecular recognition processes from experimental data. *Anal. Biochem.* 577 117–134. 10.1016/j.ab.2019.02.031 30849378

[B83] Ramasubbu N. Ragunath C. Sundar K. Mishra P. J. Gyemant G. Kandra L. (2005). Structure-function relationships in human salivary α-amylase: role of aromatic residues. *Biologia* 60 47–56.

[B84] Reetz M. T. (2001). Combinatorial and evolution-based methods in the creation of enantioselective catalysts. *Angew. Chem., Int. Ed.* 40 284–310. 10.1002/1521-3773(20010119)40:2¡284::aid-anie284¿3.0.co;2-n 11180317

[B85] Reetz M. T. Daligault F. Brunner B. Hinrichs H. Deege A. (2004). Directed evolution of cyclohexanone monooxygenases: enantioselective biocatalysts for the oxidation of prochiral thioethers. *Angew. Chem. Int. Ed. Engl.* 43 4078–4081. 10.1002/anie.200460311 15300700

[B86] Reyes-Turcu F. E. Ventii K. H. Wilkinson K. D. (2009). Regulation and cellular roles of ubiquitin-specific deubiquitinating enzymes. *Annu. Rev. Biochem.* 78 363–397. 10.1146/annurev.biochem.78.082307.091526 19489724 PMC2734102

[B87] Rohatgi N. Gudmundsson S. Rolfsson O. (2015). Kinetic analysis of gluconate phosphorylation by human gluconokinase using isothermal titration calorimetry. *FEBS Lett.* 589 3548–3555. 10.1016/j.febslet.2015.10.024 26505675

[B88] Seethala R. Menzel R. (1997). A homogeneous, fluorescence polarization assay for Src-family tyrosine kinases. *Anal. Biochem.* 253 210–218. 10.1006/abio.1997.2365 9367505

[B89] Shu Q. Frieden C. (2005). Relation of enzyme activity to local/global stability of murine Adenosine Deaminase: 19F NMR Studies. *J. Mol. Biol.* 345 599–610. 10.1016/j.jmb.2004.10.057 15581901

[B90] Siddiqui K. S. Ertan H. Charlton T. Poljak A. Daud Khaled A. K. Yang X. (2014). Versatile peroxidase degradation of humic substances: use of isothermal titration calorimetry to assess kinetics, and applications to industrial wastes. *J. Biotechnol.* 178 1–11. 10.1016/j.jbiotec.2014.03.002 24631722

[B91] Sigurskjold B. W. (2000). Exact analysis of competition ligand binding by displacement isothermal titration calorimetry. *Anal. Biochem.* 277 260–266. 10.1006/abio.1999.4402 10625516

[B92] Spencer S. D. Raffa R. B. (2004). Isothermal titration calorimetric study of RNase-A kinetics (cCMP → 3′-CMP) involving end-product inhibition. *Pharm. Res.* 21 1642–1647. 10.1023/b:pham.0000041460.78128.0f 15497691

[B93] Stroberg W. Schnell S. (2016). On the estimation errors of KM and V from time-course experiments using the Michaelis–Menten equation. *Biophys. Chem.* 219 17–27. 10.1016/j.bpc.2016.09.004 27677118

[B94] Su H. Xu Y. (2018). Application of ITC-based characterization of thermodynamic and kinetic association of ligands with proteins in drug design. *Front. Pharmacol.* 9:1133. 10.3389/fphar.2018.01133 30364164 PMC6193069

[B95] Sun Y. Cheng J. (2002). Hydrolysis of lignocellulosic materials for ethanol production: a review. *Bioresour. Technol.* 83 1–11. 10.1016/s0960-8524(01)00212-7 12058826

[B96] Szabó K. Kandra L. Gyémánt G. (2019). Studies on the reversible enzyme reaction of rabbit muscle glycogen phosphorylase b using isothermal titration calorimetry. *Carbohydr. Res.* 477 58–65. 10.1016/j.carres.2019.03.014 31005807

[B97] TA (2019). *Microcalorimetry: Itc & Dsc.* New Castle, DE: TA Instruments.

[B98] Tauran Y. Anjard C. Kim B. Rhimi M. Coleman A. W. (2014). Large negatively charged organic host molecules as inhibitors of endonuclease enzymes. *Chem. Commun.* 50 11404–11406. 10.1039/c4cc04805a 25126898

[B99] Tellinghuisen J. (2008). Isothermal titration calorimetry at very low c. *Anal. Biochem.* 373 395–397. 10.1016/j.ab.2007.08.039 17920027

[B100] Todd M. J. Gomez J. (2001). Enzyme kinetics determined using calorimetry: a general assay for enzyme activity? *Anal. Biochem.* 296 179–187. 10.1006/abio.2001.5218 11554713

[B101] Transtrum M. K. Hansen L. D. Quinn C. (2015). Enzyme kinetics determined by single-injection isothermal titration calorimetry. *Methods* 76 194–200. 10.1016/j.ymeth.2014.12.003 25497059

[B102] Turner P. Mamo G. Karlsson E. N. (2007). Potential and utilization of thermophiles and thermostable enzymes in biorefining. *Microb. Cell Fact.* 6:9. 10.1186/1475-2859-6-9 17359551 PMC1851020

[B103] Upadhyay L. S. B. (2012). Urease inhibitors: a review. *Indian J. Biotechnol.* 11 381–388.

[B104] van Spronsen F. J. Hoeksma M. Reijngoud D.-J. (2009). Brain dysfunction in phenylketonuria: Is phenylalanine toxicity the only possible cause? *J. Inherit. Metab. Dis.* 32:46. 10.1007/s10545-008-0946-2 19191004

[B105] Vander Meulen K. A. Butcher S. E. (2012). Characterization of the kinetic and thermodynamic landscape of RNA folding using a novel application of isothermal titration calorimetry. *Nucl. Acids Res.* 40 2140–2151. 10.1093/nar/gkr894 22058128 PMC3300012

[B106] Velazquez-Campoy A. Freire E. (2006). Isothermal titration calorimetry to determine association constants for high-affinity ligands. *Nat. Protoc.* 1 186–191. 10.1038/nprot.2006.28 17406231

[B107] Velázquez-Campoy A. López-Mayorga O. Cabrerizo-Vílchez M. (1999). Determination of the rigorous transfer function of an isothermal titration microcalorimeter with peltier compensation. *J. Ther. Anal. Calorim.* 57 343–359.

[B108] Wagner K. M. McReynolds C. B. Schmidt W. K. Hammock B. D. (2017). Soluble epoxide hydrolase as a therapeutic target for pain, inflammatory and neurodegenerative diseases. *Pharmacol. Ther.* 180 62–76. 10.1016/j.pharmthera.2017.06.006 28642117 PMC5677555

[B109] Wang F.-Q. Xie H. Chen W. Wang E.-T. Du F.-G. Song A.-D. (2013). Biological pretreatment of corn stover with ligninolytic enzyme for high efficient enzymatic hydrolysis. *Bioresour. Technol.* 144 572–578. 10.1016/j.biortech.2013.07.012 23896439

[B110] Wang Q. He Y. Lu R. Wang W.-M. Yang K.-W. Hai M. F. (2018). Thermokinetic profile of NDM-1 and its inhibition by small carboxylic acids. *Biosci. Rep.* 38:BSR20180244. 10.1042/BSR20180244 PMC5897741 29507059

[B111] Wang W.-J. Wang Q. Zhang Y. Lu R. Zhang Y.-L. Yang K.-W. (2017). Characterization of β-lactamase activity using isothermal titration calorimetry. *Biochim. Biophys. Acta* 1861 2031–2038. 10.1016/j.bbagen.2017.04.011 28454737

[B112] Wang Y. Guan J. Di Trani J. M. Auclair K. Mittermaier A. K. (2019). Inhibition and activation of kinases by reaction products: a reporter-free assay. *Anal. Chem.* 91 11803–11811. 10.1021/acs.analchem.9b02456 31426630

[B113] Willemoës M. Sigurskjold B. W. (2002). Steady-state kinetics of the glutaminase reaction of CTP synthase from Lactococcus lactis. *Eur. J. Biochem.* 269 4772–4779. 10.1046/j.1432-1033.2002.03175.x 12354108

[B114] Wiseman T. Williston S. Brandts J. F. Lin L.-N. (1989). Rapid measurement of binding constants and heats of binding using a new titration calorimeter. *Anal. Biochem.* 179 131–137. 10.1016/0003-2697(89)90213-3 2757186

[B115] Zebisch M. Krauss M. Schäfer P. Sträter N. (2012). Crystallographic evidence for a domain motion in rat nucleoside triphosphate diphosphohydrolase (NTPDase) 1. *J. Mol. Biol.* 415 288–306. 10.1016/j.jmb.2011.10.050 22100451

[B116] Zhang Y. Doruker P. Kaynak B. Zhang S. Krieger J. Li H. (2020). Intrinsic dynamics is evolutionarily optimized to enable allosteric behavior. *Curr. Opin. Struct. Biol.* 62 14–21. 10.1016/j.sbi.2019.11.002 31785465 PMC7250723

[B117] Zhang Y.-J. Wang W.-M. Oelschlaeger P. Chen C. Lei J.-E. Lv M. (2018). Real-time monitoring of NDM-1 activity in live bacterial cells by isothermal titration calorimetry: a new approach to measure inhibition of antibiotic-resistant bacteria. *ACS Infect. Dis.* 4 1671–1678. 10.1021/acsinfecdis.8b00147 30383355

[B118] Zheng C. J. Han L. Y. Yap C. W. Ji Z. L. Cao Z. W. Chen Y. Z. (2006). Therapeutic targets: progress of their exploration and investigation of their characteristics. *Pharmacol. Rev.* 58 259–279. 10.1124/pr.58.2.4 16714488

