# Supplementary material for: Enzyme Kinetics by Isothermal Titration Calorimetry: Allostery, Inhibition, and Dynamics
Source: Front Mol Biosci. 2020 Oct 19;7:583826. doi: 10.3389/fmolb.2020.583826 (PMC7604385; doi:10.3389/fmolb.2020.583826)
Supplement: Supplementary file 1 [file Data_Sheet_1.PDF]

Supplemental Material for:

Enzyme kinetics by isothermal titration calorimetry: allostery, inhibition, and dynamics

Yun Wang[1], Guanyu Wang[1], Nicolas Moitessier[1], Anthony Mittermaier[1]

[1]Department of Chemistry, McGill University, Montreal, Quebec, Canada

Supplementary Table 1. ITC Enzyme Kinetics Publications

Enzyme Class	Reaction	Enzyme	Organism	Reference	Advantage of ITC	Method
**Hydrolase**						
**Esterase**	hydrolysis of ester bond	Phosphotriesterase (OpdA)	*Agrobacterium radiobacter*	(1)	Traditional uses chromogenic Modified substrate.	Multiple injection; Single substrate injection; Single enzyme injection
		Glycerophospodiesterase (GpdQ)	*Enterobacter aerogenes*	(1)	Same as above	Multiple injection; Single substrate injection
		Cyclic nucleotide diesterase (Rv0805)	*Mycobacterium tuberculosis*	(1)	Same above	Multiple injection; Single substrate injection
		Phosphodiesterase (PDE1)	*Bos taurus*	(2,3)		Multiple injection
		alkaline phosphatase	*Bos taurus*	(4)		others
		RNases (ribonucleases)	*Bos taurus*	(5,6)		Single substrate injection;<br>Modified single substrate injection
		Acetylcholinesterase (AChE)	*Electrophorus electricus*	(7,8)	Neither the product nor substrate of AChE are suitable for direct spectrophotometric evaluation.	Multiple injection; Single substrate injection
		sulfatases	*Patella vulgate*	(4)		others
**Glycosylase**	hydrolysis of glycosyl compounds	Pullulanase	*Klebsiella pneumoniae*	(9-11)	Entangled alginate medium and immobilized, insoluble support matrix.	Single enzyme injection
		exo-polygalacturonases (PGs)	*Rhizopus oryzae*	(12)	The using of reducing sugar s (e.g. dinitrosalicylic acid, Somogyi–Nelson) may overestimate or underestimate reducing sugars.	Multiple injection; Single substrate injection
		nucleoside hydrolase (MtIAGU-NH)	*Mycobacterium tuberculosis*	(13)		Single enzyme injection

		α-Amylase	*Drosophila melanogaster; Homo sapiens; Sus scrofa*	(14-16)	Conventional α-Amylase activity s use a Modified substrate (e.g. chromophore-containing (CNP) maltoheptaose), which may influence the binding and catalysis mechanisms.	Single enzyme injection
		sialidase	*Arthrobacter ureafaciens*	(17)		Multiple injection
		Invertase	*Saccharomyces cerevisiae*	(18)	Membrane immobilized enzyme.	Modified single substrate injection
		Chitinases	*Arthrobacter sp. TAD20; Serratia marcescens; Serratia marcescens*	(19,20)	Insoluble substrate.	Single enzyme injection
		β-glucosidase (BG)	*Pyrococcus furiosus; Thermobifida fusca; Prunis dulcus*	(21-23)	Immobilized enzyme.	Multiple injection; Single substrate injection
		xylanase	*Aspergillus aculeatus*	(24)	Chemical-reducing-end sugar s are not always reliable.	Multiple injection
		endo-Glucanases (EGs)	*Trichoderma reesei*	(25)	The conventional uses a Modified substrate.	Single substrate injection
		Cellulases	*Hypocrea jecorina; Trichoderma reesei; Aspergillus niger;*	(26,27)	No alternative simple and quantitative to characterize a saccharification of cellulosic biomass.	Single substrate injection
		cellobiohydrolase (Cel6A)	*Hypocrea jecorina*	(28)	The use of chromogenic substrates or modified oligosaccharides do not reflect the enzymatic activity of complex substrates. ITC uses a native substrate (e.g. soluble,	Multiple injection; Single substrate injection

					insoluble and complex substrate).	
**Epoxide hydrolase**	hydrolysis of trans-substituted epoxides	epoxide hydrolase (hsEH)	*Homo sapiens*	(29)	Conventional uses LC-MS/MS	Single substrate injection;<br>Modified single substrate injection
**Acid anhydride hydrolases**	hydrolysis of acid anhydride	Nucleoside triphosphate diphosphohydrolases (NTPDases)	*Rattus norvegicus*	(30)		Multiple injection
		Lupin Diadenosine 5',5-P1,P4-Tetraphosphate Hydrolase(Ap4A)	*Lupinus angustifolius*	(31)		Multiple injection;<br>Single substrate injection
**Proteases / Peptidases**	Hydrolysis of peptide bonds	Trypsin	*Bos Taurus*	(32-37)	Conventional real-time s use Modified chromogenic substrates.	Multiple injection;<br>Single substrate injection;<br>Modified single substrate injection<br>Others (ERM)
		Nattokinase (NK)	*Bacillus subtilis*	(38)		Modified single substrate injection
		Prolyl carboxypeptidase (PRCP)	*Homo sapiens*	(39)		Multiple injection
		prolyl oligopeptidase (POP)	*Homo sapiens*	(37,40)		Modified Multiple injection;<br>Single substrate injection;<br>Others (ERM)
		Pepsin	*Sus scrofa*	(41)		Multiple injection;<br>Single substrate injection
		d,d-dipeptidase enzyme VanX	*Enterococcus faecalis; vancomycin-resistant Enterococci*	(42)	Conventional uses capillary electrophoresis. In vivo application of ITC.	Single substrate injection

**Amido-hydrolase**	hydrolysis of amide or amine bonds	β-lactamase	*Halophile Chromohalobacter sp. 560; Pseudomonas aeruginosa; Escherichia coli; Staphylococcus aureus; Acinetobacter baumannii*	(43-47)	In vivo ITC application.	Multiple injection; Single substrate injection
		Urease	*Sporosarcina pasteurii*	(48,49)		Multiple injection
		Maleamate Amidohydrolase (NicF)	*Bordetella bronchiseptica*	(50)		Multiple injection
**deaminase**	Removal of an amino group	dCMP deaminase	*Schistosoma mansoni*	(51)		Multiple injection
**Transferases**						
**Phospho-transferase (Kinase)**	transfer of phosphate groups	Gluconokinase	*Homo sapiens*	(52)	Conventional uses coupled enzymes.	Multiple injection
		Hexokinase (HK)	*Saccharomyces cerevisiae*	(53-55)	The use of labeled substrates caused a large variation of K_m for glucose and fructose(56).	Multiple injection; Single substrate injection
		Arginine kinase	*Litopenaeus vannamei*	(57)		Multiple injection
		aminoglycoside 3'-phosphotransferase-IIIa (APH (3')-IIIa)	*Enterococcus faecalis*	(58)	ITC allows real-time measurement of ADP interactions (unlike coupled)	Modified single substrate injection
		pantothenate kinases (*Ec*PanK, *Pa*PanK)	*Pseudomonas aeruginosa, Escherichia coli*	(58)	Same as above	Modified single substrate injection
		protein kinase C	*Rattus norvegicus*	(59)		Modified single substrate injection
		pyruvate kinase	*Oryctolagus Cuniculus*	(60,61)	Conventional uses coupled enzymes.	Single enzyme injection
		Taurocyamine kinase (PsTK)	*Phytophthora sojae*	(62)		Multiple injection

**Glycosyl-transferase**	formation of glycosidic linkages	glycogen phosphorylase (GP)	*Oryctolagus Cuniculus*	(63)	Custom-ITC based method directly monitored reaction kinetics in both glycogen synthesis and phosphorolysis directions.	Single enzyme injection
		Levansucrase (SacB)	*Bacillus subtilis*	(64)		Single substrate injection
**Oxidoreductases**						
	redox reaction	versatile peroxidase (VP)	*Bjerkandera adjusta*	(65,66)	Complex humic substances substrates.	Single substrate injection
		Putrescine oxidase (PUO)	*Micrococcus rubens*	(67)	Immobilized enzyme	Single substrate injection
		dihydrofolate reductase (DHFR)	*Schistosoma mansoni*	(68)		Multiple injection
		recombinant human flavincontaining monooxygenase 3 (hFMO3)	*Homo sapiens*	(69)		Multiple injection; Single substrate injection
		diaphorase	*Clostridium kluyveri*	(4)		others
		laccase	*Trametes versicolor*	(4)		others
		phenylalanine hydroxylase (PHA)	*Homo sapiens*	(70)		Multiple injection; Single enzyme injection
		dihydroorotate dehydrogenase (TcDHODH)	*Trypanosoma cruzi*	(71)	ITC employs native substrate.	Multiple injection
**Lyase**						
	elimination reaction	Adenylosuccinate lyase (SmADSL)	*Schistosoma mansoni*	(72)		Multiple injection
		Acetolactate synthase	*unknown*	(55)		Multiple injection
		Heparin lyase	*Flavobacterium . heparinum*	(55)		Multiple injection
		Rubisco	*Rhodospirillum . rubrum*	(55)		Multiple injection
**Ligases**						

	Covalently links two substrates	CTP synthase	*Lactococcus lactis*	(73)		Multiple injection
		pyruvate carboxylase	*Bos taurus*	(55)		Multiple injection
**Isomerases**						
		Chaperonin GroEL	*Escherichia coli*	(55)		Multiple injection

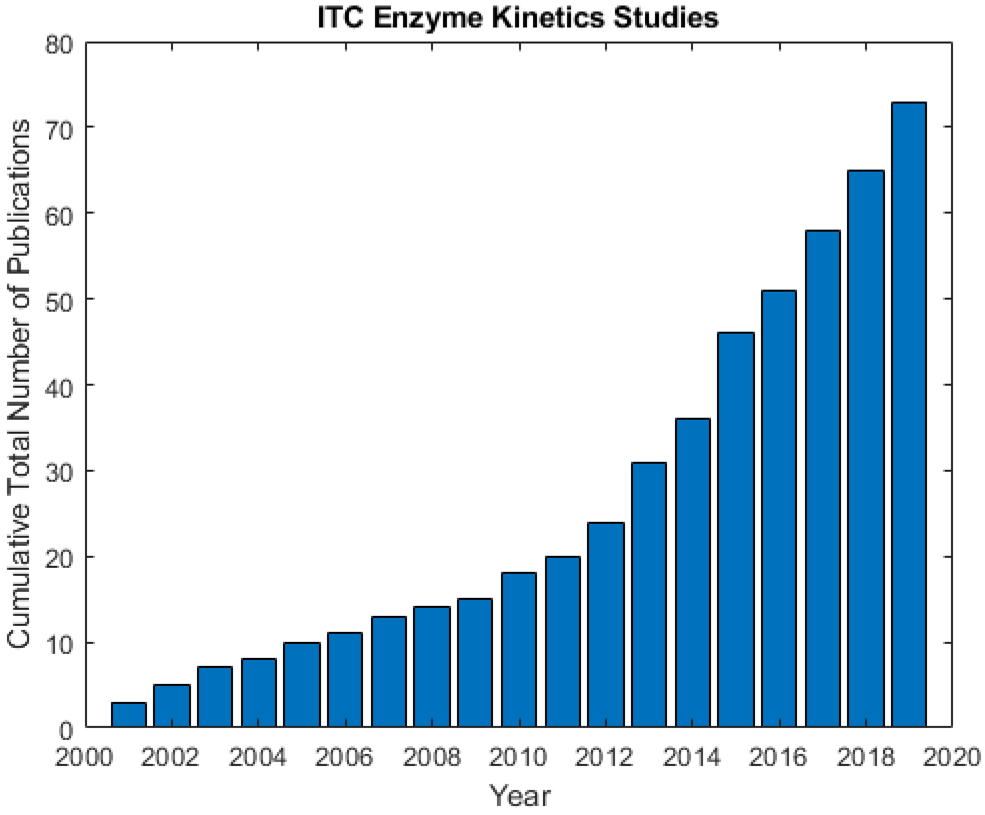

Supplemental Figure 1. Cumulative graph of ITC enzyme kinetics studies by year.

References.

1. Pedroso, M.M., Ely, F., Lonhienne, T., Gahan, L.R., Ollis, D.L., Guddat, L.W. and Schenk, G. (2014) Determination of the catalytic activity of binuclear metallohydrolases using isothermal titration calorimetry. *JBIC Journal of Biological Inorganic Chemistry*, **19**, 389-398.
2. Brulfert, F., Safi, S., Jeanson, A., Foerstendorf, H., Weiss, S., Berthomieu, C., Sauge-Merle, S. and Simoni, É. (2017) Enzymatic activity of the CaM-PDE1 system upon addition of actinyl ions. *Journal of Inorganic Biochemistry*, **172**, 46-54.

3. Stateva, S., Salas, V., Benaim, G., Menendez, M., Solís, D. and Villalobo, A. (2015) Characterization of Phospho-(Tyrosine)-Mimetic Calmodulin Mutants. *PloS one*, **10**, e0120798.
4. Honarmand Ebrahimi, K., Hagedoorn, P.-L., Jacobs, D. and Hagen, W.R. (2015) Accurate label-free reaction kinetics determination using initial rate heat measurements. *Scientific Reports*, **5**, 16380.
5. Spencer, S.D. and Raffa, R.B. (2004) Isothermal Titration Calorimetric Study of RNase-A Kinetics (cCMP → 3'-CMP) Involving End-Product Inhibition. *Pharmaceutical Research*, **21**, 1642-1647.
6. Cai, L., Cao, A. and Lai, L. (2001) An Isothermal Titration Calorimetric Method to Determine the Kinetic Parameters of Enzyme Catalytic Reaction by Employing the Product Inhibition as Probe. *Anal. Biochem.*, **299**, 19-23.
7. de Almeida Neves, P.A., Silva, E. and Beirão, P. (2017) Microcalorimetric Study of Acetylcholine and Acetylthiocholine Hydrolysis by Acetylcholinesterase. *Advances in Enzyme Research*, **2017**, 1-12.
8. Draczkowski, P., Tomaszuk, A., Halczuk, P., Strzemski, M., Matosiuk, D. and Jozwiak, K. (2016) Determination of affinity and efficacy of acetylcholinesterase inhibitors using isothermal titration calorimetry. *Biochimica et Biophysica Acta (BBA) - General Subjects*, **1860**, 967-974.
9. Ali, G., Dulong, V., Gasmi, S.N., Rihouey, C., Picton, L. and Le Cerf, D. (2015) Covalent immobilization of pullulanase on alginate and study of its hydrolysis of pullulan. *Biotechnology Progress*, **31**, 883-889.
10. Ali, G., Rihouey, C., Le Cerf, D. and Picton, L. (2013) Effect of carboxymethyl groups on degradation of Modified pullulan by pullulanase from Klebsiella pneumoniae. *Carbohydrate Polymers*, **93**, 109-115.
11. Ali, G., Rihouey, C., Larreta-Garde, V., Le Cerf, D. and Picton, L. (2013) Molecular Size Characterization and Kinetics Studies on Hydrolysis of Pullulan by Pullulanase in an Entangled Alginate Medium. *Biomacromolecules*, **14**, 2234-2241.
12. Mertens, J.A. (2013) Kinetic Properties of Two Rhizopus Exo-polygalacturonase Enzymes Hydrolyzing Galacturonic Acid Oligomers Using Isothermal Titration Calorimetry. *Applied Biochemistry and Biotechnology*, **170**, 2009-2020.
13. Wink, P.L., Sanchez Quitian, Z.A., Rosado, L.A., Rodrigues, V.d.S., Petersen, G.O., Lorenzini, D.M., Lipinski-Paes, T., Saraiva Macedo Timmers, L.F., de Souza, O.N., Basso, L.A. *et al.* (2013) Biochemical characterization of recombinant nucleoside hydrolase from Mycobacterium tuberculosis H37Rv. *Archives of Biochemistry and Biophysics*, **538**, 80-94.
14. Kaeswurm, J.A.H., Claasen, B., Fischer, M.-P. and Buchweitz, M. (2019) Interaction of Structurally Diverse Phenolic Compounds with Porcine Pancreatic α-Amylase. *Journal of Agricultural and Food Chemistry*, **67**, 11108-11118.
15. Lehoczki, G., Szabó, K., Takács, I., Kandra, L. and Gyémánt, G. (2016) Simple ITC method for activity and inhibition studies on human salivary α-amylase. *Journal of Enzyme Inhibition and Medicinal Chemistry*, **31**, 1648-1653.
16. Commin, C., Aumont-Nicaise, M., Claisse, G., Feller, G. and Da Lage, J.-L. (2013) Enzymatic characterization of recombinant α-amylase in the Drosophila melanogaster species subgroup: is there an effect of specialization on digestive enzyme? *Genes Genet. Syst.*, **88**, 251-259.
17. Chen, C.-S. and Lee, W.-J. (2018) Study of the Enzymatic Activity of Arthrobacter ureafaciens Neuraminidase by Isothermal Titration Calorimetry. *Journal of the Chinese Chemical Society*, **65**, 271-275.
18. Mason, M., Scampicchio, M., Quinn, C.F., Transtrum, M.K., Baker, N., Hansen, L.D. and Kenealey, J.D. (2018) Calorimetric Methods for Measuring Stability and Reusability of Membrane Immobilized Enzymes. *Journal of Food Science*, **83**, 326-331.

19. Lonhienne, T., Baise, E., Feller, G., Bouriotis, V. and Gerday, C. (2001) Enzyme activity determination on macromolecular substrates by isothermal titration calorimetry: application to mesophilic and psychrophilic chitinases. *Biochimica et Biophysica Acta (BBA) - Protein Structure and Molecular Enzymology*, **1545**, 349-356.
20. Krokeide, I.-M., Eijsink, V.G.H. and Sørlie, M. (2007) Enzyme for chitinase catalyzed hydrolysis of tetra-N-acetylchitotetraose by isothermal titration calorimetry. *Thermochimica Acta*, **454**, 144-146.
21. Park, S.H., Park, K.H., Oh, B.C., Alli, I. and Lee, B.H. (2011) Expression and characterization of an extremely thermostable β-glycosidase (mannosidase) from the hyperthermophilic archaeon Pyrococcus furiosus DSM3638. *New Biotechnology*, **28**, 639-648.
22. Jeoh, T., Baker, J.O., Ali, M.K., Himmel, M.E. and Adney, W.S. (2005) β-d-Glucosidase reaction kinetics from isothermal titration microcalorimetry. *Analytical Biochemistry*, **347**, 244-253.
23. Henzler, K., Haupt, B. and Ballauff, M. (2008) Enzymatic activity of immobilized enzyme determined by isothermal titration calorimetry. *Analytical Biochemistry*, **378**, 184-189.
24. Baumann, M.J., Murphy, L., Lei, N., Krogh, K.B.R.M., Borch, K. and Westh, P. (2011) Advantages of isothermal titration calorimetry for xylanase kinetics in comparison to chemical-reducing-end s. *Analytical Biochemistry*, **410**, 19-26.
25. Murphy, L., Cruys-Bagger, N., Damgaard, H.D., Baumann, M.J., Olsen, S.N., Borch, K., Lassen, S.F., Sweeney, M., Tatsumi, H. and Westh, P. (2012) Origin of initial burst in activity for Trichoderma reesei endo-glucanases hydrolyzing insoluble cellulose. *J. Biol. Chem.*, **287**, 1252-1260.
26. Murphy, L., Bohlin, C., Baumann, M.J., Olsen, S.N., Sørensen, T.H., Anderson, L., Borch, K. and Westh, P. (2013) Product inhibition of five Hypocrea jecorina cellulases. *Enzyme and Microbial Technology*, **52**, 163-169.
27. Murphy, L., Borch, K., McFarland, K.C., Bohlin, C. and Westh, P. (2010) A calorimetric for enzymatic saccharification of biomass. *Enzyme and Microbial Technology*, **46**, 141-146.
28. Murphy, L., Baumann, M.J., Borch, K., Sweeney, M. and Westh, P. (2010) An enzymatic signal amplification system for calorimetric studies of cellobiohydrolases. *Analytical Biochemistry*, **404**, 140-148.
29. Abis, G., Pacheco-Gómez, R., Bui, T.T.T. and Conte, M.R. (2019) Isothermal Titration Calorimetry Enables Rapid Characterization of Enzyme Kinetics and Inhibition for the Human Soluble Epoxide Hydrolase. *Analytical Chemistry*, **91**, 14865-14872.
30. Zebisch, M., Krauss, M., Schäfer, P. and Sträter, N. (2012) Crystallographic Evidence for a Domain Motion in Rat Nucleoside Triphosphate Diphosphohydrolase (NTPDase) 1. *Journal of Molecular Biology*, **415**, 288-306.
31. Branson, K.M., Mertens, H.D.T., Swarbrick, J.D., Fletcher, J.I., Kedzierski, L., Gayler, K.R., Gooley, P.R. and Smith, B.J. (2009) Discovery of Inhibitors of Lupin Diadenosine 5′,5′′′-P1,P4-Tetraphosphate Hydrolase by Virtual Screening. *Biochemistry*, **48**, 7614-7620.
32. Maximova, K., Wojtczak, J. and Trylska, J. (2019) Enzyme kinetics in crowded solutions from isothermal titration calorimetry. *Analytical Biochemistry*, **567**, 96-105.
33. Di Trani, J.M., Moitessier, N. and Mittermaier, A.K. (2018) Complete Kinetic Characterization of Enzyme Inhibition in a Single Isothermal Titration Calorimetric Experiment. *Anal. Chem. (Washington, DC, U. S.)*, **90**, 8430-8435.
34. Transtrum, M.K., Hansen, L.D. and Quinn, C. (2015) Enzyme kinetics determined by single-injection isothermal titration calorimetry. *Methods*, **76**, 194-200.

35. Maximova, K. and Trylska, J. (2015) Kinetics of trypsin-catalyzed hydrolysis determined by isothermal titration calorimetry. *Analytical Biochemistry*, **486**, 24-34.
36. Aguirre, C., Condado-Morales, I., Olguin, L.F. and Costas, M. (2015) Isothermal titration calorimetry determination of individual rate constants of trypsin catalytic activity. *Analytical Biochemistry*, **479**, 18-27.
37. Di Trani, J.M., Moitessier, N. and Mittermaier, A.K. (2017) Measuring Rapid Time-Scale Reaction Kinetics Using Isothermal Titration Calorimetry. *Analytical Chemistry*, **89**, 7022-7030.
38. Weng, M., Deng, X., Bao, W., Zhu, L., Wu, J., Cai, Y., Jia, Y., Zheng, Z. and Zou, G. (2015) Improving the activity of the subtilisin nattokinase by site-directed mutagenesis and molecular dynamics simulation. *Biochemical and Biophysical Research Communications*, **465**, 580-586.
39. Kehoe, K., Van Elzen, R., Verkerk, R., Sim, Y., Van der Veken, P., Lambeir, A.-M. and De Meester, I. (2016) Prolyl carboxypeptidase purified from human placenta: its characterization and identification as an apelin-cleaving enzyme. *Biochimica et Biophysica Acta (BBA) - Proteins and Proteomics*, **1864**, 1481-1488.
40. Di, T.J.M., De, C.S., O'Leary, R., Plescia, J., do, N.C.J., Moitessier, N., Mittermaier, A.K. and do, N.C.J. (2018) Rapid measurement of inhibitor binding kinetics by isothermal titration calorimetry. *Nat Commun*, **9**, 893.
41. Luo, Q., Chen, D., Boom, R.M. and Janssen, A.E.M. (2018) Revisiting the enzymatic kinetics of pepsin using isothermal titration calorimetry. *Food Chemistry*, **268**, 94-100.
42. Lv, M., Zhang, Y.-J., Zhou, F., Ge, Y., Zhao, M.-H., Liu, Y. and Yang, K.-W. (2019) Real-time monitoring of D-Ala-D-Ala dipeptidase activity of VanX in living bacteria by isothermal titration calorimetry. *Analytical Biochemistry*, **578**, 29-35.
43. Antunes, V.U., Llontop, E.E., Vasconcelos, F.N.d.C., Lopez de los Santos, Y., Oliveira, R.J., Lincopan, N., Farah, C.S., Doucet, N., Mittermaier, A. and Favaro, D.C. (2019) Importance of the β5-β6 Loop for the Structure, Catalytic Efficiency, and Stability of Carbapenem-Hydrolyzing Class D β-Lactamase Subfamily OXA-143. *Biochemistry*, **58**, 3604-3616.
44. Zhang, Y.-J., Wang, W.-M., Oelschlaeger, P., Chen, C., Lei, J.-E., Lv, M. and Yang, K.-W. (2018) Real-Time Monitoring of NDM-1 Activity in Live Bacterial Cells by Isothermal Titration Calorimetry: A New Approach To Measure Inhibition of Antibiotic-Resistant Bacteria. *ACS Infectious Diseases*, **4**, 1671-1678.
45. Wang, Q., He, Y., Lu, R., Wang, W.-M., Yang, K.-W., Fan, Hai M., Jin, Y. and Blackburn, G.M. (2018) Thermokinetic profile of NDM-1 and its inhibition by small carboxylic acids. *Bioscience Reports*, **38**.
46. Wang, W.-J., Wang, Q., Zhang, Y., Lu, R., Zhang, Y.-L., Yang, K.-W., Lei, J.-E. and He, Y. (2017) Characterization of β-lactamase activity using isothermal titration calorimetry. *Biochimica et Biophysica Acta (BBA) - General Subjects*, **1861**, 2031-2038.
47. Arai, S., Yonezawa, Y., Okazaki, N., Matsumoto, F., Shibazaki, C., Shimizu, R., Yamada, M., Adachi, M., Tamada, T., Kawamoto, M. *et al.* (2015) Structure of a highly acidic β-lactamase from the moderate halophile Chromohalobacter sp. 560 and the discovery of a Cs(+)-selective binding site. *Acta Crystallogr D Biol Crystallogr*, **71**, 541-554.
48. Mazzei, L., Cianci, M., Benini, S., Bertini, L., Musiani, F. and Ciurli, S. (2016) Kinetic and structural studies reveal a unique binding mode of sulfite to the nickel center in urease. *Journal of Inorganic Biochemistry*, **154**, 42-49.

49. Benini, S., Cianci, M., Mazzei, L. and Ciurli, S. (2014) Fluoride inhibition of Sporosarcina pasteurii urease: structure and thermodynamics. *JBIC Journal of Biological Inorganic Chemistry*, **19**, 1243-1261.
50. Kincaid, V.A., Sullivan, E.D., Klein, R.D., Noel, J.W., Rowlett, R.S. and Snider, M.J. (2012) Structure and Catalytic Mechanism of Nicotinate (Vitamin B3) Degradative Enzyme Maleamate Amidohydrolase from Bordetella bronchiseptica RB50. *Biochemistry*, **51**, 545-554.
51. Scortecci, J.F., Serrão, V.H.B., Cheleski, J., Torini, J.R., Romanello, L., DeMarco, R. and D'Muniz Pereira, H. (2017) Spectroscopic and calorimetric s reveal dependence on dCTP and two metals (Zn2++Mg2+) for enzymatic activity of Schistosoma mansoni deoxycytidylate (dCMP) deaminase. *Biochimica et Biophysica Acta (BBA) - Proteins and Proteomics*, **1865**, 1326-1335.
52. Rohatgi, N., Gudmundsson, S. and Rolfsson, O. (2015) Kinetic analysis of gluconate phosphorylation by human gluconokinase using isothermal titration calorimetry. *FEBS Lett.*, **589**, 3548-3555.
53. Olsen, S.N. (2006) Applications of isothermal titration calorimetry to measure enzyme kinetics and activity in complex solutions. *Thermochim. Acta*, **448**, 12-18.
54. Bianconi, M.L. (2003) Calorimetric determination of thermodynamic parameters of reaction reveals different enthalpic compensations of the yeast hexokinase isozymes. *J. Biol. Chem.*, **278**, 18709-18713.
55. Todd, M.J. and Gomez, J. (2001) Enzyme kinetics determined using calorimetry: A general for enzyme activity? *Anal. Biochem.*, **296**, 179-187.
56. Bianconi, M.L. (2007) Calorimetry of enzyme-catalyzed reactions. *Biophys. Chem.*, **126**, 59-64.
57. Lopez-Zavala, A.A., Sotelo-Mundo, R.R., Hernandez-Flores, J.M., Lugo-Sanchez, M.E., Sugich-Miranda, R. and Garcia-Orozco, K.D. (2016) Arginine kinase shows nucleoside diphosphate kinase-like activity toward deoxythymidine diphosphate. *J. Bioenerg. Biomembr.*, **48**, 301-308.
58. Wang, Y., Guan, J., Di Trani, J.M., Auclair, K. and Mittermaier, A.K. (2019) Inhibition and Activation of Kinases by Reaction Products: A Reporter-Free . *Analytical Chemistry*, **91**, 11803-11811.
59. Streeter, J., Schickling, B.M., Jiang, S., Stanic, B., Thiel, W.H., Gakhar, L., Houtman, J.C.D. and Miller, F.J., Jr. (2014) Phosphorylation of Nox1 regulates association with NoxA1 activation domain. *Circ Res*, **115**, 911-918.
60. Lonhienne, T.G.A. and Winzor, D.J. (2002) Calorimetric Demonstration of the Potential of Molecular Crowding To Emulate the Effect of an Allosteric Activator on Pyruvate Kinase Kinetics. *Biochemistry*, **41**, 6897-6901.
61. Lonhienne, T.G.A., Reilly, P.E.B. and Winzor, D.J. (2003) Further evidence for the reliance of catalysis by rabbit muscle pyruvate kinase upon isomerization of the ternary complex between enzyme and products. *Biophysical Chemistry*, **104**, 189-198.
62. Palmer, A., Begres, B.N., Van Houten, J.M., Snider, M.J. and Fraga, D. (2013) Characterization of a putative oomycete taurocyamine kinase: Implications for the evolution of the phosphagen kinase family. *Comparative Biochemistry and Physiology Part B: Biochemistry and Molecular Biology*, **166**, 173-181.
63. Szabó, K., Kandra, L. and Gyémánt, G. (2019) Studies on the reversible enzyme reaction of rabbit muscle glycogen phosphorylase b using isothermal titration calorimetry. *Carbohydrate Research*, **477**, 58-65.
64. Méndez-Lorenzo, L., Porras-Domínguez, J.R., Raga-Carbajal, E., Olvera, C., Rodríguez-Alegría, M.E., Carrillo-Nava, E., Costas, M. and López Munguía, A. (2015) Intrinsic Levanase Activity of Bacillus subtilis 168 Levansucrase (SacB). *PloS one*, **10**, e0143394-e0143394.

65. Ertan, H., Siddiqui, K.S., Muenchhoff, J., Charlton, T. and Cavicchioli, R. (2012) Kinetic and thermodynamic characterization of the functional properties of a hybrid versatile peroxidase using isothermal titration calorimetry: Insight into manganese peroxidase activation and lignin peroxidase inhibition. *Biochimie*, **94**, 1221-1231.
66. Siddiqui, K.S., Ertan, H., Charlton, T., Poljak, A., Daud Khaled, A.K., Yang, X., Marshall, G. and Cavicchioli, R. (2014) Versatile peroxidase degradation of humic substances: Use of isothermal titration calorimetry to assess kinetics, and applications to industrial wastes. *Journal of Biotechnology*, **178**, 1-11.
67. Henao-Escobar, W., Domínguez-Renedo, O., Alonso-Lomillo, M.A., Cascalheira, J., Dias-Cabral, A. and Arcos‐Martínez, M. (2014) Characterization of a Disposable Electrochemical Biosensor Based on Putrescine Oxidase from Micrococcus rubens for the Determination of Putrescine. *Electroanalysis*, **27**.
68. Serrão, V.H.B., Romanello, L., Cassago, A., de Souza, J.R.T., Cheleski, J., DeMarco, R., Brandão-Neto, J. and Pereira, H.D.M. (2017) Structure and kinetics s of recombinant Schistosoma mansoni dihydrofolate reductase. *Acta Tropica*, **170**, 190-196.
69. Catucci, G., Sadeghi, S.J. and Gilardi, G. (2019) A direct time-based ITC approach for substrate turnover measurements demonstrated on human FMO3. *Chemical Communications*, **55**, 6217-6220.
70. Pey, A.L. and Martinez, A. (2005) The activity of wild-type and mutant phenylalanine hydroxylase and its regulation by phenylalanine and tetrahydrobiopterin at physiological and pathological concentrations: An isothermal titration calorimetry study. *Molecular Genetics and Metabolism*, **86**, 43-53.
71. Cheleski, J., Wiggers, H.J., Citadini, A.P., da Costa Filho, A.J., Nonato, M.C. and Montanari, C.A. (2010) Kinetic mechanism and catalysis of Trypanosoma cruzi dihydroorotate dehydrogenase enzyme evaluated by isothermal titration calorimetry. *Analytical Biochemistry*, **399**, 13-22.
72. Romanello, L., Serrão, V.H.B., Torini, J.R., Bird, L.E., Nettleship, J.E., Rada, H., Reddivari, Y., Owens, R.J., DeMarco, R., Brandão-Neto, J. *et al.* (2017) Structural and kinetic analysis of Schistosoma mansoni Adenylosuccinate Lyase (SmADSL). *Molecular and Biochemical Parasitology*, **214**, 27-35.
73. Willemoës, M. and Sigurskjold, B.W. (2002) Steady-state kinetics of the glutaminase reaction of CTP synthase from Lactococcus lactis. *European Journal of Biochemistry*, **269**, 4772-4779.
